# Deleting Death and Dialysis: Conservative Care of Cardio-Vascular Risk and Kidney Function Loss in Chronic Kidney Disease (CKD)

**DOI:** 10.3390/toxins10060237

**Published:** 2018-06-12

**Authors:** Raymond Vanholder, Steven Van Laecke, Griet Glorieux, Francis Verbeke, Esmeralda Castillo-Rodriguez, Alberto Ortiz

**Affiliations:** 1 Nephrology Section, Department of Internal Medicine, Ghent University Hospital, 9000 Ghent, Belgium; steven.vanlaecke@ugent.be (S.V.L.); griet.glorieux@ugent.be (G.G.); francis.verbeke@ugent.be (F.V.); 2 Department of Nephrology and Hypertension, IIS-Fundacion Jimenez Diaz UAM, 28040 Madrid, Spain; ecastillo@quironsalud.es (E.C.-R.); aortiz@fjd.es (A.O.)

**Keywords:** chronic kidney disease, CKD, mortality, outcomes, uremia, medication, lifestyle, diet, prevention, cardiovascular disease

## Abstract

The uremic syndrome, which is the clinical expression of chronic kidney disease (CKD), is a complex amalgam of accelerated aging and organ dysfunctions, whereby cardio-vascular disease plays a capital role. In this narrative review, we offer a summary of the current conservative (medical) treatment options for cardio-vascular and overall morbidity and mortality risk in CKD. Since the progression of CKD is also associated with a higher cardio-vascular risk, we summarize the interventions that may prevent the progression of CKD as well. We pay attention to established therapies, as well as to novel promising options. Approaches that have been considered are not limited to pharmacological approaches but take into account lifestyle measures and diet as well. We took as many randomized controlled hard endpoint outcome trials as possible into account, although observational studies and post hoc analyses were included where appropriate. We also considered health economic aspects. Based on this information, we constructed comprehensive tables summarizing the available therapeutic options and the number and kind of studies (controlled or not, contradictory outcomes or not) with regard to each approach. Our review underscores the scarcity of well-designed large controlled trials in CKD. Nevertheless, based on the controlled and observational data, a therapeutic algorithm can be developed for this complex and multifactorial condition. It is likely that interventions should be aimed at targeting several modifiable factors simultaneously.

## 1. Introduction

The clinical picture accompanying both acute kidney injury (AKI) and chronic kidney disease (CKD) affects almost every organ system [1,2] and results in a progressively worsening clinical condition that, if untreated, ends in coma and death (End-Stage Kidney Disease—ESKD). Since the forties of previous century, renal replacement therapies (dialysis or transplantation) prolonged survival chances, however, without entirely abolishing the complication profile or correcting the cardio-vascular risk. Only more recently, the focus shifted to the pre-dialysis stage in attempts to prevent progression of kidney disease and its most devastating complications, especially cardio-vascular damage. 

### 1.1. Traditional and Non-Traditional Risk Factors of Renal Cardiovascular Disease

Cardio-vascular disease (CVD) in CKD patients is characterized by immunity-driven inflammatory changes which set the stage for vessel wall stiffening, and extensive arteriopathy and cardiomyopathy, leading to heart failure, arrhythmia and cardiac arrest [1]. Risk factors for those cardiac and vascular disorders span from traditional causes to an expanding list of disease-peculiar features and surrogate outcomes such as endothelial dysfunction [3].

The gap between the predictive value of traditional risk factors and cardio-vascular mortality in CKD [4] is to a substantial extent captured by the indicators of kidney dysfunction such as estimated glomerular filtration rate (eGFR) and albuminuria [5]. These factors are also essential in the definition of the stages of CKD that should be taken into account when studies are conducted in pre-specified stages (Table 1). Factors related to declining kidney function, such as subclinical volume expansion and uremic solute retention play a role in this process. Many uremic retention solutes have been associated with the causes of cardio-vascular damage, such as inflammation, oxidative stress, macrophage activation and infiltration, endothelial dysfunction, thrombogenesis, arterial calcification, or osteodystrophy. However, other factors also play a role in this process such as hypertension, dyslipidemia, electrolyte disturbances and disruption of hormonal axes, homeostatic mechanisms and glucose handling. Together, all of these elements define the mechanisms that have been the focus of research which over the last decades emanated in the development of therapeutic options that result in or have the potential to result in the improvement of outcomes of CKD.

Although dialysis and transplantation extend life expectation of uremic patients, overall cardio-vascular mortality remains markedly higher than in age-matched populations with normal kidney function [6,7], and this discrepancy starts already during the early stages of CKD [8,9]. In this publication, we review the therapeutic options to cope with these problems.

### 1.2. Concept of This Publication

There were, at least up till very recently, less controlled hard outcome studies in the area of kidney disease than in that of other non-communicable (chronic) diseases [10], and such studies often found negative or contradictory results [3]. This might be attributed to the complex and multifactorial nature of the disease (differences in primary causes, genetic predisposition, metabolism and uremic retention pattern), making it difficult to compose large patient groups with a uniform patho-physiological background. In addition, the multilayered patho-physiology makes single therapeutic options to be often overwhelmed by other factors, blurring eventual effects on outcomes. Therefore, the present narrative review will also be supported by study types other than randomized controlled trials (RCTs) focusing on hard outcomes.

**Figure 1:**
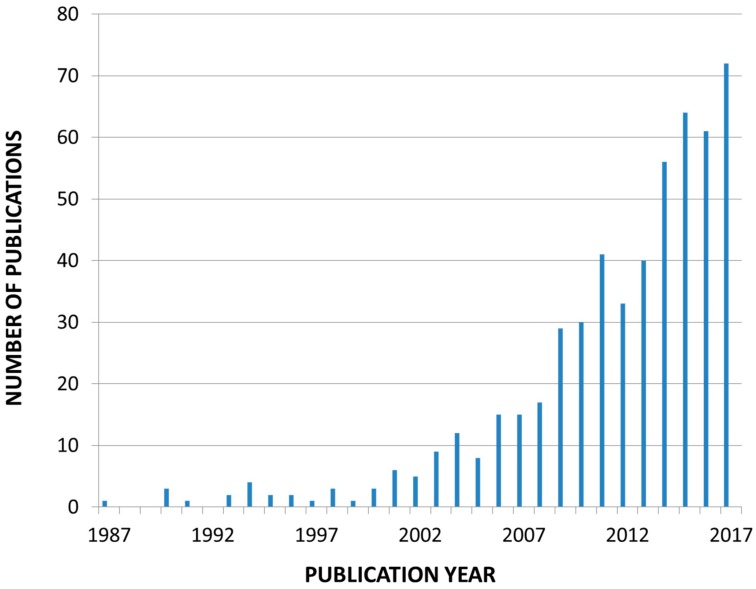
Number of publications retrieved for this paper per publication year.

The growing interest in evaluating the measures to prevent CVD in CKD and/or progression of CKD can be captured by analyzing the publication years of the articles contained in the reference list of this paper, which shows an exponential rise over time (Figure 1).

### 1.3. Search Strategy

In what follows, treatment aimed at modifying cardio-vascular risk will be discussed in broad terms considering a number of underlying mechanism(s). In contrast to previous reviews that often focused on specific causative mechanisms, we offer a kaleidoscopic view of the many different aspects possibly improving the outcomes in CKD. 

Literature was searched up to 1 February 2018 with classical engines such as Pubmed, Web of Science, Google Scholar and Cochrane Library. Search terms related to the main topics of this publication were used (e.g., “metabolic bone disease” or “inflammation”—the full list of possible terms can be deduced from the summary tables included in this publication) and “chronic kidney disease”, “chronic renal failure” or “CKD” and “treatment”, “therapy”, “outcomes”, “morbidity” or “mortality”. Recent RCTs or large observational studies on hard outcomes were retained as much as possible, however, if these were absent, smaller and surrogate outcome studies were considered. We also searched the reference lists of the retrieved articles for other relevant publications. If data were available, we paid attention to health aspects as well as to health-economic and socio-economic aspects. The measures slowing down or preventing the development of cardio-vascular disease were considered, as well as those slowing down progression of CKD, given the suggestive data in favor of an association between level of GFR and cardio-vascular events [5,8,9]. The data on prevention of CKD progression will be discussed together with the cardio-vascular outcomes in the respective sections and the summary tables. Only Anglo-Saxon literature was retrieved.

As this review is narrative, the literature database may be incomplete, but we made efforts to include the most relevant articles as much as possible. 

A separate search was conducted with the name of some specific individual uremic compounds (e.g., uric acid, homocysteine) and the other terms mentioned above to retrieve studies on therapies aiming to reduce the concentration of those compounds. The selection of those solutes was based on a previous review by the authors describing evidence for toxicity of a large array of uremic toxins [11].

This paper is conceived as an update and extension of a previous review [1], accounting for the ample additional information that has been provided for the time being. The present review is limited to conservative treatment, i.e., lifestyle measures, diet and pharmaceutical therapies. Extracorporeal renal replacement therapies will be discussed in a separate article of this special issue. Specific therapies targeted at particular renal conditions (e.g., tolvaptan for adult polycystic kidney disease or preventive measures for kidney stones) were not considered. In addition, AKI and its prevention, although definitely linked to CKD [12] and thus a likely cause of cardio-vascular complications [13], was not considered and the reader is referred to specific reviews on this topic [14].

## 2. Lifestyle Correction

In researching for cardio-vascular and kidney protection in CKD, the emphasis is often laid on pharmacological interventions with at times, sophisticated drugs, of which the benefit is not necessarily unequivocally proven. However, some general lifestyle measures may also offer benefit, while involving minimal societal investment apart from educational initiatives and essentially organizational or regulatory efforts [15].

### 2.1. Cessation of Smoking

Smoking has been strongly linked to cardio-vascular disease through a multitude of patho-physiologic mechanisms including inflammation, thrombogenesis, oxidative stress, enhanced production of advanced glycation end-products (AGEs) and oxidation of low density lipoprotein (LDL) [16,17,18]. Of note, all of these elements have also been linked to the cardio-vascular damage of uremia [1,19]. Thus, smoking conceptually enhances the deleterious cardio-vascular effect of uremia and vice versa. Smoking has also been associated with glomerulosclerosis in animal experiments [20]. In previous clinical studies, smoking was linked to CKD and its progression to ESKD, with a more pronounced impact with intense smoking or if it was associated with other conditions affecting cardio-vascular status, such as hypertension or diabetes [21,22,23,24]. According to a recent systematic review of observational studies, present but also former smoking was independently associated with the development of incident CKD [25]. Smoking has also been associated in non-diabetics with the development of nodular glomerulosclerosis reminiscent of diabetic nephropathy [26]. Maternal smoking during pregnancy was associated with an increased risk for childhood proteinuria [27]. Although inevitably prone to residual confounding, all of these studies suggest a deleterious role of smoking in CKD.

The impact of smoking on mortality in specific CKD populations has only rarely been studied, but at least in one observational study in Japanese CKD patients, smoking increased all-cause and cardio-vascular mortality [28]. In a post hoc analysis of the Study of Heart and Renal Protection (SHARP) trial, smoking was linked to increased cardio-vascular and overall mortality, but not to progression of CKD [29].

The socio-economic impact of smoking and passive smoking is considered substantial and expands far beyond direct health-related aspects, including workplace absenteeism, early retirement, the costs of cleaning up smokers’ garbage and restoring damage related to smoking [30]. According to an assessment undertaken in Germany, the smoking costs continued to rise per decade at least up till 2006 [31], and the health service savings of smoking cessation have been stressed [32]. However, in a number of analyses, no economic benefit was suggested for some European countries, due to the incremental costs from longer survival in non-smokers [32,33]. In these studies, however, essential factors such as productivity and quality of life were often neglected, and the impact of passive smoking on overall health and of active smoking on hypertension, CKD or ESKD were not included [32,33].

In brief, based on observational data, smoking seems to be associated with the progression of kidney disease and also likely to mortality in CKD (Table 2, part A). However, it should be realized that it will probably never be possible to organize an RCT regarding this issue. Concerning the societal cost, data are contradictory but often blurred by the fact that some key consequences such as hypertension or CKD were not taken into account (Table 2, part A). In addition, another factor that is insufficiently considered is the health economic benefit of tobacco taxes. It has been modeled that increasing cigarette cost by 10% in all EU countries would not only decrease tobacco consumption and subsequent death toll but would also make available extra funds to cover smoking related health costs [34]. 

### 2.2. Exercise

Physical inactivity in the general population has been associated with factors impacting cardio-vascular risks such as higher body weight and blood pressure, decreased insulin sensitivity and an unfavorable balance between high density lipoprotein (HDL) and low density lipoprotein (LDL) [35]. Physical inactivity is also considered to be the major cause of mortality, affecting not only cardio-vascular status but also the propensity for malignancy [35]. In subjects with a known cardio-vascular disease, cardiac rehabilitation had a positive effect on outcomes [36,37,38], although these positive results may partly be skewed due to selection bias. Older people without limitations of physical exercise capacity survive longer [39]. It has been recommended that strategies to prevent cardio-vascular disease, including stimulation of physical activity, should be organized at a primary health promotion level, and this appeared to have a socio-economic and health-economic benefit, especially if direct supervision is not required [40]. Cost-utility was often equivalent to that of pharmaceutical intervention [41,42].

Although no hard outcome studies could be retrieved in a narrative review on exercise in non-dialyzed CKD, several studies suggest a positive impact on cardio-vascular risk factors, such as hypertension, inflammation and oxidative stress [43]. Due to the lack of specific guidelines for CKD, it was suggested to follow the recommendations for older people and other chronic diseases [43]. Also in the hemodialysis population, exercise training has been suggested to be beneficial, at least for well-being [44]. In an RCT assessing caloric restriction, exercise or the combination of both in CKD patients, each of both interventions, as well as their combination, led to a decrease in inflammatory parameters [45].

In an observational post-hoc analysis of the Ongoing Telmisartan Alone and Combination With Ramipril Global Endpoint Trial (ONTARGET), daily physical activity was associated with a lower risk in developing CKD and for mortality [46,47].

A meta-analysis of RCTs considering the entire spectrum of CKD found a large number of studies showing a benefit but with a high risk of bias, and all limited to a short follow-up period. The favorable impact was essentially limited to physical fitness and quality of life [48], while another meta-analysis confirmed these data but also pointed to an impact on cardio-vascular function [49]. Benefits, if any, were better established in the dialysis population than those not on dialysis [48], but the risk for selection bias was also highest in the dialyzed population.

In brief, the currently available data is suggestive of a positive impact of exercise on clinical condition (Table 2, part A), mortality and progression of CKD although most data come from observational studies and/or surrogate outcome studies. More detailed research is needed before this suggestion can be confirmed.

### 2.3. Reduction of Overweight and Obesity

In the general population, obesity may affect the cardio-vascular system through its negative impact on many well-known risk factors such as dyslipidemia, glucose intolerance, inflammation, thrombogenesis and sleep apnea as well as through many other mechanisms, resulting in multiple cardiac complications such as coronary heart disease, heart failure, arrhythmia and sudden death, and reduced life expectancy [50,51]. Obesity has also been linked to subclinical cardio-vascular disease [52]. The health economic burden of obesity and overweight in countries such as Switzerland and Canada has been estimated to 2.3 to 4.1% of overall health care expenditure, with a more or less equal share of respective costs for overweight and obesity [53,54]. In a health economic assessment of the Belgian population on the potential effect of body weight reduction, a decrease of body mass index (BMI) by 1 kg/m^2^ resulted in substantial cost savings, irrespective of gender, and in both obese and overweight subjects [55]. On the other hand, in a study based on the Dutch population, lifetime health expenditure was lower in the obese than in the healthy-living population due to the shorter life expectancy of obese people [33]. 

In Sweden, the rate of adult obesity increased from 5% to over 11% from 1980 to 2010, an evolution that ran in parallel to a rise in the consumption of ultra-processed food products, especially soda, snacks and candies [56].

In two meta-analyses, combating obesity by bariatric and other surgical intervention as compared to lifestyle/medical intervention induced more remission of type 2 diabetes, better glycemic control and lowering of Hemoglobin A1C, but both studies concluded that hard outcome data were missing [57,58]. In contrast, studies with the anti-obesity drugs orlistat and sibutramine on blood pressure generated contradictory results with only a marginal decrease in blood pressure for orlistat that was limited to non-diabetics [59]. In one meta-analysis, long-term pharmaceutical treatment of obesity resulted in an improvement of several cardio-vascular risk factors, but likewise, hard outcome data were missing [60].

After hypertension and together with high blood glucose, obesity is the most important risk factor for mortality burden due to cardio-vascular disease, CKD, and diabetes [61]. It is linked to CKD by causing sodium retention, sympathetic and Renin Angiotensin Aldosterone System (RAAS) activation, renal hyperfiltration, inflammation and hypertension [62].

In a case-control study based on the Kidney Early Evaluation Program (KEEP), obesity was not associated with the development of CKD [63]. In contrast, in a large cohort considering over 3 million US veterans with eGFR > 60 mL/min/1.73 m^2^, a high BMI was associated with a rapid loss of kidney function, especially with increasing age [64]. Two meta-analyses in CKD concluded that there was a positive effect of weight loss intervention on blood pressure, proteinuria and albuminuria [65,66], while GFR was normalized particularly in cases of hyperfiltration and morbid obesity [65]. In an RCT in CKD patients, weight loss reduced pro-inflammatory parameters [45]. In an analysis of more than 2000 adults (79% women) who underwent bariatric surgery, an improvement in CKD risk category was seen in a large proportion of patients, even up to seven years after the intervention, and especially in the higher risk categories [67].

Of note, in the hemodialysis population, an obesity paradox has been observed with better survival for obese patients [68], analogous to similar phenomena observed in the context of hypertension and dyslipidemia, but probably confounded by worse outcomes of the malnourished segment of the dialysis population [68]. The obesity paradox also applies to other chronic conditions such as cardio-vascular disease, congestive heart failure and hypertension [51,69], but has in part been attributed to the use of BMI as an indicator of obesity, rather than other markers that are more consistently related with negative hard endpoint outcomes such as waist circumference, which show a straightforward positive relation to hard outcomes over all ranges [69].

If an intentional weight loss is planned, e.g., to allow enrolment on the transplantation waiting list, the benefit should be balanced with the risk of protein wasting as a consequence of caloric restriction, particularly at the later CKD stages [1].

In general, the impact of obesity, overweight, and weight loss on mortality in CKD seems less straightforward than for smoking and exercise (Table 2, part A), with the exception of surrogate cardio-vascular outcomes, but data may have partly been confounded by the use of less reliable parameters of obesity and by the counterbalancing negative impact of malnutrition on outcomes. On the other hand, data on surrogate outcomes, as well as the progression of CKD, tended to improve with obesity reduction.

### 2.4. Improving Environmental Factors

Environmental pollution is a lifestyle factor that has been considered insufficiently as an overall and cardio-vascular risk factor in the context of CKD, although environment has been linked to cardio-vascular disease [70]. Some environmental factors, such as heavy metals, high ambient temperatures, environmentally linked infections such as malaria or leptospirosis and industrial chemicals have all been related to CKD or AKI development [71]. Incident CKD and progression to ESKD have been related to particulate matter air pollution [72], although there remains a potential risk of confounding due to socio-economic factors (i.e., as housing is cheaper in polluted areas, these are usually inhabited by people with less financial resources who are also more prone to develop non-communicable diseases including CKD). According to most experts, a recent epidemic of CKD among field workers in Sri Lanka, India, and Central America, is due to environmental factors, although there is no agreement about the real cause [73]. Some hypothesized a role for glyphosate, possibly in combination with hard water [73].

The only way to prevent these environmental factors to play a role is to limit exposure but to the best of our knowledge, there are no data on what the impact would be of such interventions, and by definition, it will be difficult to collect such data in a controlled way.

### 2.5. Conclusions

Although indirect arguments and surrogate outcome studies support the impact of lifestyle factors on CKD, the conditions leading to CKD and cardio-vascular disease in CKD, these aspects, in general, have received little attention, as compared to pharmacological intervention (Table 2, part A). Furthermore, hard endpoint outcome RCTs and health-economic analyses on lifestyle in CKD are often lacking or remain inconclusive. For the progression of kidney disease, there are more data available in which smoking, lack of exercise and obesity seem to play important roles. Data on environmental effect are largely deficient. Lack of evidence seems more attributable to the scarcity of data than to negative results. Even if it is acknowledged that designing lifestyle studies in CKD is not always simple, efforts should be made to increase the number and the quality of such studies in CKD.

## 3. Dietary Interventions

### 3.1. Protein Restriction

The concept that protein restriction might protect against progression of CKD became accepted well before the end of the previous century. Next to a reduction of glomerular filtration pressure [74], also other metabolic effects with the potential to make cardio-vascular outcomes better, such as improvement of calcium/phosphate metabolism or insulin resistance have been put forward [75].

However, a number of RCTs on protein restriction showed no benefit. In the Modification of Diet in Renal Disease (MDRD) trial, low protein diet vs. standard, as well as very low protein diet plus keto-analogues vs. low protein diet did not significantly delay the decline of GFR, be it at the borderline of significance for the study arm comparing very low protein diet plus keto-analogues to low protein diet [76]. Also, later follow-up studies of the MDRD trial showed no effect, but imposing dietary thresholds had been discontinued after the conclusion of the proper trial [77,78]. Of note, in the MDRD study, the targeted protein intake was reached with neither low protein diet nor very low protein diet [76], underscoring the difficulty of bringing CKD patients to lower their protein intake as part of a dietary intervention. On the other hand, as CKD advances, patients tend to spontaneously lower their protein intake as part of uremic anorexia [79], which is an additional confounder and a potential source of muscular and protein energy wasting. 

Additionally, a smaller Italian RCT comparing low protein diet with moderate protein diet showed no difference in the risk to die or to start dialysis [80]. Another RCT, however, showed an advantage on the progression of CKD with very low vegetable protein diet plus keto-analogues compared to low protein diet [81]. In a longitudinal study, very low protein intake with keto-analogues favorably influenced blood pressure [82]. A case control study also showed a reduction in acid load, together with blood pressure reduction [83]. 

One meta-analysis on diabetic nephropathy showed a significant effect of low protein diet on GFR but not on proteinuria, but the quality of the studies included was considered low [84]. Another meta-analysis comparing restricted protein diet (both low and very low protein diet together) to higher protein intake in non-diabetic adults showed a slower progression of CKD [85] that, however, was essentially attributable to the subgroup with very low protein diet [86]. Study quality was considered low [86]. No hard outcome studies or quality of life studies were found [86]. Also in children, no impact on the progression of CKD could be observed [87].

One matter of concern with low protein diet is the risk of protein energy wasting (PEW) [88]. Although this possibility has been acknowledged, especially among the elderly [86], few data are available. In one meta-analysis in children with CKD, no parameters conforming to PEW were found with low protein diet, but data were limited to one study under carefully supervised settings, and the authors expressed concerns that low protein diet could lead to poor growth [87]. In a longitudinal study in adults, a worsening of nutritional status was demonstrated [89], but an RCT could find no significant difference in nutritional parameters between very low protein diet plus keto-analogues and conventional low protein diet [80].

Perhaps the lack of impact of classical low protein diet is more related to the kind of protein that is provided than to the quantity of protein ingested. In the RCT showing a benefit on the progression for very low protein diet plus keto-analogues, the diet was vegetable-based [81]. An observational study disclosed a relationship between red meat intake and microalbuminuria [90]. Red meat is also the source of trimethylamine-N-oxide (TMAO), an agent linked to cardio-vascular damage in some studies [91] and retained in CKD [92]. One of the precursors, carnitine, is not only present in red meat but it is also used as food supplement and thus potentially deleterious by promoting atherosclerosis, especially in CKD [93]. Of note, the role of TMAO as the cardio-vascular risk factor is not entirely straightforward. Fish, e.g., has a high TMAO content but is not to increase cardio-vascular risk at all [94].

Compared to standard animal protein intake, soy protein intake had a beneficial effect on cardio-renal indices and inflammatory parameters in patients with diabetic nephropathy [95]. In stage G 4 hypertensive CKD, a fruit and vegetable diet reduced metabolic acidosis [96], and a similar alkalinizing effect was also demonstrated in a large CKD population, together with a suppression of fibroblast growth factor-23 (FGF-23) [97], although the compensatory decrease in protein intake might as such have caused a decrease in systemic acidification. In addition, in observational analyses, dietary fiber ingestion also resulted in better glycemic control and more favorable cardio-vascular risk factors in diabetic CKD patients [98] and decreased inflammation in CKD of all causes [99]. Finally, vegetarian or fiber diet decreased the concentration of protein bound uremic toxins [100,101,102]. The Mediterranean diet which combines fiber intake to other potentially beneficiary nutrients (potassium, polyunsaturated fatty acids, olive oil) has been linked to better preservation of kidney function and survival in some studies [103,104]. All these findings suggest that the potential benefits of changing protein intake may be attributable to quality changes rather than to quantity changes of protein sources. 

In conclusion, the role of protein intake and restriction in CKD remains contradictory and controversial (Table 2, part B). Hard outcomes and quality of life data are largely missing. With regards to the progression of CKD, the most convincing results have been obtained with very low protein diet associated to keto-analogues (Table 2, part B), whereby lack of long term adherence, higher cost or decrease in quality of life, however, may offset a potential benefit. The risk of malnutrition remains an overall matter of concern. In essence, unless very low protein diet plus keto-analogues are prescribed, a protein intake comparable to the one recommended in the general population can be prescribed in CKD patients not on dialysis, inclusive transplants (0.8 g/kg/day or even lower—0.6–0.8 g/kg/day) (Table 3) [1,105].

On dialysis, additional catabolism occurs in response to chronic inflammation, surgical interventions, recurrent sepsis and nutrient losses during the dialysis procedure. Thus, when dialysis starts, protein intake should be increased to more than 1.0 g protein/kg/day in hemodialysis patients and 1.2 g/kg/day in peritoneal dialysis (PD) (Table 3) [1,106], although it may be difficult for patients to make this sudden dramatic shift in dietary attitude.

### 3.2. Sodium Restriction

The role of sodium intake in the general population remains controversial. A number of observational studies demonstrated a J-shaped curve with higher cardio-vascular morbidity and mortality with an intake both below and above the standard of 4–6 g/d NaCl (1.6–2.4 g/d of sodium) [107,108] or even a worse cardio-vascular outcome only with low sodium intake [109]. Possibly worse outcomes with high sodium intakes in the non-CKD population are essentially related to the hypertensive subgroup [108]. Likewise, in a study with diabetics, mortality followed a J-shaped curve, while evolution to ESKD was most likely with low sodium, not with a high sodium diet [110]. Although most studies adjusted for potential confounders, such as poor general health associated with low sodium intake, residual confounding cannot be excluded. Another problem is that the variable reflecting sodium intake most often is not sodium intake per se but a surrogate, daily sodium excretion, which is prone to sampling error, as sodium intake may change from day to day. Moreover, only one measurement at baseline is generally entered into the analysis. In contrast, and because of its pertinent link to increased blood pressure in hypertensive patients, sodium moderation remains being advocated especially in the hypertensive population [111]. 

Additionally, in CKD, observational studies gave quite contradictory results. In one study, high sodium intake was associated with the progression of kidney failure [90], but two other studies showed no associations [112,113]. In one study with peritoneal dialysis patients, low sodium intake based upon dietary records was even predictive of higher mortality risk, a finding that possibly was related to low overall nutrient intake not limited to sodium ingestion alone and subsequent protein energy wasting [114]. On the other hand, an additive effect of salt restriction to the Renin-Angiotensin System (RAS) inhibition was observed with regards to renal and cardio-vascular protection [115,116] whereas high dietary salt (>14 g/d) blunted the protective impact of RAS inhibition on proteinuria and incidence of ESKD [117].

Results with regards to blood pressure in CKD are more straightforward. In a short-term RCT, salt restriction resulted in a decrease of blood pressure [118,119], as well as of extracellular fluid volume and albuminuria [118] and N-terminal pro-brain natriuretic peptide (NT-pro-BNP) [120] which, however, was accompanied by a short-term decrease in eGFR [120]. Of note, such a short-term decrease after initiation of therapy was also registered with RAS inhibition and protein restriction. In these two cases, the early decline in eGFR was followed by a subsequent nephroprotective effect [76,121]. Thus, the finding of an early eGFR decline after intervention should not necessarily be considered as a negative sign.

In line with the above data, two systematic reviews found no RCTs showing a positive impact of low sodium diet on hard outcomes [86,122], the only definite positive effect being the one on hypertension [122]. The published information is, however, extremely heterogeneous [123]. One of the reasons for these negative outcome results may be that one of these reviews found no studies with a running time above 6 months [86], which makes the finding of a difference at hard endpoint analyses rather unlikely. 

In conclusion, the current recommended sodium intake in CKD of 5–6 g/d NaCl (Table 3) [1] is based on the lowest zone of negative hard outcomes in the general population studies and the apparent capacity of sodium restriction to decrease surrogate outcomes such as hypertension and fluid overload in CKD. Effect on the progression of CKD is controversial except in patients on angiotensin converting enzyme inhibitors (ACEi) and angiotensin receptor blockers (ARB) (Table 2, part B). The lack of hard outcome data in CKD makes it impossible to draw more firm conclusions, and thus there is an absolute need for further study in this domain.

### 3.3. Potassium Rich Diet

Possibly, rather than by sodium intake per se, the pathogenesis of hypertension is driven by the interaction between sodium and potassium intake [111]. In general, unless extra potassium salts are ingested, daily potassium load is defined by fruit and vegetable intake, which often is low in CKD, especially in dialysis patients [124]. An observational study found no relationship between sodium intake and renal outcomes, but there was a straightforward relationship between increasing potassium intake and favorable evolution of kidney function [113]. Increasing vegetable intake was linked to less evolution to CKD [47], although such observational studies might be prone to bias by reverse causation, as patients with more advanced CKD are often advised to lower potassium intake. The potassium-rich Dietary Approaches to Stop Hypertension (DASH) diet had an additive blood pressure lowering effect on top of sodium restriction [119].

One matter of concern with fruit and vegetable diet in CKD is the risk of hyperkalemia [125]. Unfortunately, data on the safety of such diet in advanced CKD remain scant. In the study of the effects of the DASH diet [119], the effects in CKD were the same as in the overall group of participants but serum potassium and clinical adverse events were not considered in this analysis [126], and none of the included patients suffered from CKD stage G 4–5 [126]. In a study assessing the impact of fruit and vegetable diet on metabolic acidosis in CKD stage G 4, none of the 36 patients receiving the diet developed an increase in serum potassium over a 1-year period but baseline serum potassium > 4.6 mmol/L was an exclusion criterion [96]. In general, it seems a logical approach to be careful with stimulating potassium intake in patients with advanced CKD and to take into account the potassium concentration and general clinical status in each specific individual with CKD, before stimulating potassium intake. Whether administering potassium binders would enable a more relaxed attitude in this regard needs to be proven and will be discussed more extensively in the section on RAAS-inhibition.

In summary, if hyperkalemia can be avoided, stimulating potassium intake, or rather tipping over the balance of sodium vs. potassium intake in favor of potassium might have a clinical advantage (Table 2, part B), although most studies are observational and based on surrogate outcomes. In addition, it is not always clear whether the advantage is related to the dietary potassium or the fiber content in fruit/vegetable diet, or a combination of the two. Also, observational studies demonstrating a benefit of high potassium intake on the decline of kidney function might suffer from reverse causation. In general, it is recommended to keep potassium intake below 2.5 g/d in all CKD patients with stage G 3 or above including those on dialysis (Table 3) [1], although more liberal intake may be considered if serum potassium levels remain within acceptable range.

### 3.4. Phosphorus Restriction

The phosphorus content of the Western diet is high and in excess of natural needs, leading to deleterious compensatory changes of regulatory hormones even in the healthy population [127]. This excess is to a large extent attributable to the abundance of phosphate salts in processed food [127].

A fortiori, phosphate load is even more deleterious in CKD patients, in whom the capacity to excrete phosphate in the urine is hampered. Educating ESKD patients to avoid foods with high added phosphate content resulted in a decrease of phosphatemia [128].

In observational studies, serum phosphorus was independently linked to progression of CKD [129,130] and was suggested to counteract the renoprotective effect of ACE-inhibition [130] and very low protein diet [131]. Also, in a population with normal to moderately decreased kidney function (eGFR > 60 mL/min), the risk to reach ESKD increased with higher baseline and time averaged serum phosphate [132].

Only a few studies have assessed the impact of dietary phosphate restriction on hard outcomes. Prescription of restricted dietary phosphate in hemodialysis patients was associated with worse survival [133]. Despite the difference in survival which disappeared after adjustment for confounders, actual serum phosphate levels and degree of protein and phosphate intake were similar in individuals with prescribed phosphate restriction and those without [133]. Perhaps the negative survival outcome with phosphate restriction was attributable to the difficulty in dissociating the changes in phosphate intake from those in other essential macronutrients, as patients on restriction had poorer nutrition status indices [133]. In another observational study, phosphate restriction only had a positive impact on outcomes if combined with an increased protein intake [134].

Hence, although diet is an obvious cause of phosphate load, study data on the impact of dietary phosphate restriction on hard outcomes are conflicting and supported by only limited evidence (Table 2, part B), possibly because it is difficult to avoid a decrease of protein intake or ingestion of other essential nutrients when restricting phosphate. Pharmacological interventions to reduce serum phosphate will be discussed below.

### 3.5. Enhanced Polyunsaturated Fatty Acid (PUFA) Intake

PUFAs are most prominently present in fish but are also provided as a food supplement in the form of fish oil. Both options will be discussed together in this section.

There are a number of observational studies linking fish or PUFA intake to lowering of cardio-vascular events or mortality in the general population and patients with CKD or at risk of CKD, such as diabetics. A meta-analysis of RCTs found no relationship between fish or omega-3 fatty acid consumption and cerebro-vascular disease [135], and only the analysis of observational data showed a moderate decrease in risk with higher intakes [135]. Higher intakes of fish were also related to a lower risk of microalbuminuria in young diabetes type 1 patients [136]. In CKD, PUFAs were linked to a decrease of inflammatory parameters in a dose-dependent manner [137]. In hemodialysis patients, low red blood cell docosahexanoic acid independently predicted mortality [138,139].

Higher dietary fish intake was associated with lower mortality risk in incident dialysis patients in an observational study [140]. Similarly, eicosapentaenoic acid administration was also associated with lower mortality in hemodialysis [141].

In a meta-analysis of RCTs, fish oil supplementation suppressed the risk of arterio-venous fistula events and cardio-vascular events [142]. However, in a small RCT in hemodialysis patients, n-3 PUFAs had no impact on the primary outcome (a composite of cardio-vascular events and death) [143].

In one meta-analysis limited to studies in IgA nephropathy, no impact was seen on proteinuria, but a positive outcome was observed on the progression of CKD as defined by a doubling of serum creatinine or evolution to ESKD. However, this conclusion was based on data extracted from only two studies [144]. However, another meta-analysis merely showed opposite results with no benefit on renal function parameters but an improvement of proteinuria [145].

Some data point to a beneficial impact of PUFAs, either on cardio-vascular events or progression of CKD (Table 2, part B), but it is difficult to find a consistent trend in the findings. In addition, we considered an amalgam of studies on fish intake and several different PUFA preparations, and it is not sure whether one size fits all. Finally, bad taste and breath odor may affect the quality of life and decrease adherence to some of these preparations. Therefore, we probably need other large RCTs, including both quality of life analyses and hard outcomes before we can come to definite conclusions.

### 3.6. Sugar Intake Restriction

High sugar intake via sugared beverages has been linked to the metabolic syndrome, abdominal obesity, hypertension, albuminuria and the development and progression of CKD among different ethnicities [146,147,148]. According to a nutrient analysis that essentially focused on products made available in the USA, about 75% of processed foods contained added sugars [149], implying a solid rationale to reduce this type of inadvertent sugar intake. According to health-economic calculations, bringing down the dietary sugar intake to the recommended levels implied a substantial societal financial benefit [150].

Also, excessive fructose intake has been associated with increased blood pressure and the metabolic syndrome, independent of caloric intake, an effect linked to ATP depletion and uric acid generation [151]. In a longitudinal cross-over protocol, a switch to a low fructose diet tended to decrease blood pressure but this effect was limited to the subgroup of nocturnal blood pressure dippers [152]. Also, a decrease in inflammatory parameters was observed [152]. However, in a meta-analysis of 21 controlled trials, isocaloric high fructose diet had no impact on plasma uric acid [153].

Increased levels of advanced glycosylated end products (AGEs) in CKD have been linked to increased mortality in some [154] but not all studies [155]. Low AGE diet decreased AGE concentration in patients with CKD [156], and reduced inflammatory parameters [157]. As compared to high AGE diet, low AGE diet improved renal function and inflammatory markers in obese individuals [158].

Summarizing, restricting excess glucose intake via sugared drinks and processed foods, and reducing dietary AGE content, and possibly also fructose load, may decrease surrogate outcomes related to cardio-vascular risk, but hard evidence remains scant (Table 2, part B).

### 3.7. Correction of Intestinal Dysbiosis

Intestinal microbiota plays a role in generating certain uremic retention solutes [1]. RCTs aiming to restore intestinal symbiosis in CKD by administering prebiotics (selectively fermentable ingredients), probiotics (live biotherapeutics) or synbiotics (combination of pre- and probiotics) and to reduce circulating levels of uremic solutes, inflammation, and progression of CKD are scarce [159,160,161,162]. Specific prebiotics, such as resistant starch and gum arabic fiber, decreased serum indoxyl sulfate in hemodialysis patients [160] and urea nitrogen in CKD patients [161], while administration of the prebiotic arabinoxylan oligosaccharides to CKD patients not on dialysis did not affect microbiota derived uremic solutes, nor insulin resistance [163]. For probiotics, a recent RCT in diabetic hemodialysis patients showed beneficial effects on parameters of glucose homeostasis, and specific biomarkers of inflammation and oxidative stress [162]. Another RCT in PD patients also demonstrated a beneficial effect on pro-and anti-inflammatory cytokines, together with better preservation of residual kidney function in the group receiving probiotics [164]. Synbiotic therapy reduced levels of p-cresol/p-cresyl sulfate but not indoxyl sulfate [159,165] and slowed down the decrease in eGFR [166]. Of interest, next to a decrease in serum p-cresyl sulphate upon synbiotic, Rossi et al. revealed an alteration in stool microbiome consisting of enrichment of *Bifidobacterium* and depletion of *Ruminococcaceae* [159].

In summary, data on the effect of pre-, pro- and synbiotic therapy on hard outcomes are still scarce and not convincing (Table 2, part B). Interventions improving symbiosis need further study before their validity can be accepted. 

The intestinal sorbent AST-120 will be discussed separately below. 

### 3.8. Diet: General Conclusions

The current consensus regarding the target diet in CKD favors salt, phosphate and protein restriction, and fiber intake [105,167]. Evidence supporting these dietary options is relatively weak (Table 2, part B), partially linked to the low quality of the studies, but also to the difficulties to obtain strict adherence among study participants and the multifactorial nature of CKD with many confounders such as comorbidities and drug intake interfering with the impact of diet. Well-conducted RCTs are very much needed because of the low societal cost of diet. In addition, such approaches would help to exclude confounding by compensatorily increased intake of unhealthy nutrients if appropriate diet for one or more other food components is pursued. This would also help to distinguish and correct for adherence problems and potential negative effects of some specific dietary measures that are proper to CKD (e.g., protein malnutrition with phosphate restriction or hyperkalemia with fiber intake). It will however remain hard to control the intake of the large array of all food elements if some of them are modified by intervention.

There is a scarcity of health economic analyses (Table 2, part B), which in part is related to a lack of good hard outcome studies, which in turn, is linked to the low macro-economic interest of diet as compared to pharmaceuticals. On the other hand, the advantage on pharmaceutical intervention seems obvious because of the low societal cost of diet. However, one aspect that is often forgotten is the high costs of healthy food for the individual, which may be especially important for CKD, which is a frequent condition among people with a low income. In the last few years, there has been an increase of pressure on the production sector of processed foods and retail to reduce production and promotion of unhealthy food, although there is still much room for improvement [15] as not all involved parties modified their strategy yet, and procedures could be made more efficient, e.g., by imposing governmental regulations, rather than leaving the initiative to self-regulation, as what happens now.

A key factor of the dietary management of the uremic syndrome is “to supply enough but not too much”, as the body needs fuel but cannot handle the end-products of cellular metabolism well enough. This is particularly obvious for proteins since they have no storage system to adjust for intake fluctuations [1].

Specialized dieticians should regularly be involved in the implementation of advice to correctly educate the patient. At least three encounters per year have been suggested the first year of care. Practically, this seems workable only from CKD stage G 4 on. Twenty-four-hour urine collections allow monitoring daily protein intake (via urea measurements) and sodium intake and should be performed twice yearly to control and implement the diet [1,168], although sampling errors are prone to affect the reliability of the results.

## 4. Pharmacological Treatment

The pharmacological treatment and prevention of the cardio-vascular complications and the progression of CKD have received more and more attention over the last decade, in recognition of the important impact on health and health economy of CKD at large, and especially of ESKD [15]. In the next three sections we will first focus on antihypertensive treatment, then on other interventions than blood pressure lowering (e.g., anticoagulation or correction of acidosis) not directly aimed at influencing the activity of particular uremic toxins, and then on specific therapies coping with the action of particular uremic toxins. Next to established therapies, we will focus on emerging options, which have not always been the subject of thorough clinical analysis. 

### 4.1. Antihypertensive Treatment

#### 4.1.1. Intensive Treatment

Antihypertensive treatment in the general population reduces the incidence of death and cardio-vascular events at all risk levels and in absolute terms (number of lives saved) most of all in patients with a higher baseline risk [169]. The risk reduction gains in importance as blood pressure lowering is more substantial [169]. A meta-analysis on close to 45,000 patients in the general population showed greater vascular protection and less albuminuria in case of intensive blood pressure lowering [170].

In pre-dialysis CKD, the well-established cardio-vascular benefit of anti-hypertensive treatment has led to specific guidelines recommending target blood pressures of ≤140/90 mmHg in CKD as in the general population (≤130/80 in the presence of proteinuria) [171]. 

Several RCTs addressed the impact of intensive blood pressure control (target usually < 130/80 mmHg) as compared to standard antihypertensive treatment in non-CKD and CKD populations. In the African-American Study of Kidney Disease (AASK), in black CKD patients, intensive treatment showed no advantage on the progression of CKD or death, although subgroup analysis suggested a benefit with regard to the progression of CKD for those with increased proteinuria (≥1 g/day) [172]. In the Action to Control Cardiovascular Risk (ACCORD) study in diabetics with reasonable kidney function, no benefit on overall cardio-vascular outcome was found in the intensive treatment arm (systolic blood pressure ≤ 120 mmHg), with worsening of some kidney function parameters (rise in serum creatinine and decline of eGFR) [173]. However, the secondary outcome of stroke was lower in the intensive arm [173]. In the Systolic Blood Pressure Intervention Trial (SPRINT), enrolling non-diabetics among which also a substantial subgroup with CKD, and representing a considerable proportion of the current US population [174], targeting a systolic blood pressure below 120 mmHg in the intensive arm resulted in lowering of major cardio-vascular events and death, however, at the expense of a larger proportion of non-CKD patients developing a substantial decrease in eGFR (≥30% reduction to CKD stage G 3 or lower) [175]. Although the study cannot be defined as CKD specific [176], the subgroup with CKD also showed a similar outcome benefit, with one of the kidney function parameters (change of eGFR per year) slightly worse in the intensive group [177].

A major point of concern has been the higher incidence of AKI in the intensive treatment arm of SPRINT, in which overall tension declined rapidly in the early phase of the study [175], whereas those cases of AKI tended to occur more frequently with larger declines of blood pressure [178]. The question has been raised, however, in how far these episodes were true occurrences of injury or rather potentially reversible changes of GFR due to hemodynamic changes [179]. On the other hand, a post hoc analysis of the AASK and MDRD trials showed that an early fall of eGFR by ≥ 20% increased the risk for ESKD, irrespective of whether the treatment regime was intensive or not [180].

Another particularity of the SPRINT trial is the use of unobserved automated blood pressure measurements to avoid a white coat effect. This technique may have resulted in up to 20 mmHg lower systolic blood pressure readings compared to conventional ambulatory measurements, meaning that the arm targeting systolic tensions below 120 mmHg, in fact, should be compared to values of around 140 mmHg in other trials [181].

Several meta-analyses addressed the impact of intensive blood pressure control (target usually < 130/80 mmHg) as compared to standard antihypertensive treatment in CKD populations. A meta-analysis in non-diabetic CKD concluded that decline of kidney function could be slowed down when targeting a blood pressure between 110 and 129 mmHg systolic in the presence of proteinuria > 1 g/d, although the authors stated that reverse causation (more severe kidney disease causing higher blood pressures) could not be excluded [182]. The effect of intensive blood pressure lowering was markedly less prominent with proteinuria < 1 g/d [182]. Blood pressure lowering < 110 mmHg increased the risk of worsening kidney function [182]. Another meta-analysis showed no benefit on the progression of non-diabetic CKD and on all-cause mortality with targeting blood pressure below standard, although there was a beneficial trend for kidney function for non-blacks and patients with higher proteinuria [183], while another analysis also showed no difference in the analyzed outcomes (mortality, cardio-vascular events and progression of CKD) if targeting blood pressure below 125–130/75–80 mmHg versus below 140/90 [184]. However, the most recent meta-analysis in CKD populations showed a decrease in mortality with more intensive blood pressure control [185].

In dialysis patients, the relationship between pre- or post-dialysis blood pressure and mortality is inverse or U-shaped, which is sometimes considered a classical example of reverse causality. However, dialysis-related tension values hardly reflect true blood pressure burden in hemodialysis patients [186,187,188,189]. Out-of-dialysis systolic blood pressure, in fact, predicts a linear increase in the risk of death from 110 mmHg on, as in the general population [190].

In ESKD patients, including patients with heart failure, the use of antihypertensive drugs reduces mortality [191], but treatment should be titrated to tolerable levels, i.e., minimizing the risk of hypotension due to autonomic dysfunction or arterial stiffness, which enhances the risk for ischemic events [192]. Judicious use of anti-hypertensive drugs accounting for comorbidities and pharmacokinetic profile may reduce cardio-vascular risk in dialysis patients [193]. A recent pilot study of limited extent over short duration focused only on pre-dialysis blood pressure to compare intensive antihypertensive treatment to standard in a hemodialysed population [194]. It showed a potential safety signal by demonstrating a non-significant trend for more hospitalizations and vascular access adverse events in the intensive treatment arm, but those data were considered not important enough to withhold a more extended RCT that would be based on the same design including the use of pre-dialysis blood pressure and not out-of-dialysis blood pressure as a target parameter [194].

The cumulated data in all these studies suggest that in the general population and even in patients with non-dialyzed CKD but without diabetes, blood pressure targets might be further lowered to 130/80 mmHg (Table 4, part A). The same target had been proposed already in the KDIGO 2012 blood pressure guidelines for CKD with albuminuria/proteinuria > 30/150 mg/g urinary creatinine [195]. Blood pressure should, however, preferentially be reduced with care and gradually, with judicious consideration of the clinical condition of the patient (comorbidities, vascular stiffness, age) and the evolution of kidney function. In view of the many exclusion criteria of the SPRINT trial, of which diabetes is the main but not the only one, this care especially applies to patients at risk for major hemodynamic complications if blood pressure declines suddenly or too much. In addition, hypertension should be consistent (implying several measurements with an interval per consultation or even at more than one consultation) before treatment of any kind or intensity is launched [171]. In the dialysis population, blood pressures when not on dialysis should be considered, and lowered to standard (≤140/90 mmHg) or even below if needed. However, in this group even more than in patients not on dialysis, attention should be paid to the risk profile of the patient, authorizing higher blood pressure targets when deemed necessary in view of the patient’s clinical condition. 

#### 4.1.2. Angiotensin Converting Enzyme Inhibitors (ACEi) and Angiotensin Receptor Blockers (ARB)

Several clinical studies pointed to protection against progression of CKD (either albuminuria or parameters of glomerular filtration) by administration of ACEi [196,197,198,199,200,201,202,203]. In at least one study, these results were obtained although blood pressure reduction with ACEi was the same as in the control (non-ACEi) group [197], and protection was observed irrespective of the stage of CKD at the start of therapy, even in non-diabetics [197]. In diabetics, ACEi in normoalbuminuric patients could prevent microalbuminuria [201].

Similarly, ARB was also shown in several studies to provide nephroprotection [121,198,204,205,206,207,208], and this nephroprotection occurred independently of blood pressure lowering per se in some of these studies [121,206]. In addition, nephroprotection was provided irrespective of baseline kidney function [208]. Although it is hard to deny the impression that, compared to ACEi, ARB were proportionally less studied in non-diabetic CKD than in diabetic nephropathy, some data also refer to non-diabetics [204,209]. In addition, at least in type 2 diabetics, nephroprotection was observed even when the drug was started very early in the evolution of the disease, in patients with microalbuminuria [121] or even normoalbuminuria [206].

Of note, in a meta-analysis considering studies using both RAS inhibitors and other interventions, the mere fact of reducing proteinuria as such was also linked to preventing progression towards ESKD [210].

Single studies have paid much less attention to the cardio-vascular effect of either ACEi or ARB in CKD, but in a number of trials, no difference [200,201] or even more fatal cardio-vascular events were seen [206].

One meta-analysis focusing on diabetic CKD reported a benefit on CKD progression with ARB alone and the combination of ARB and ACEi, although with the latter intervention the advantage was offset by a borderline risk for hyperkalemia [211]. In this study, no regime offered a survival advantage [211]. Another meta-analysis, including CKD of any type, showed for both ACEi and ARB, a reduction of evolution to kidney failure and cardio-vascular events [212]. ACEi was with regards to cardio-vascular events, possibly superior to ARB [212]. This analysis did not separate diabetic from non-diabetic CKD or early from later stages. In a somewhat older meta-analysis on a limited number of substantially heterogeneous studies on non-diabetics with early CKD, no definite conclusions could be drawn [213].

In an RCT comparing an ACEi alone, an ARB alone, and a combination of both vs. placebo, both RAS inhibitors were equivalent regarding renal protection, but the combination had no added value and was associated with more adverse outcomes, especially renal impairment and hyperkalemia [214]. On the other hand, as mentioned, a meta-analysis in diabetics did not come to the same conclusion and pointed to a nephroprotective effect of the combination [211]. A recent systematic review showed a benefit for any combined RAAS inhibition (ACEi + ARB; ACEi or ARB + aldosterone blockade) vs. single RAAS blockade for overall but not cardio-vascular mortality [215]. Cardiovascular mortality at subanalysis appeared significant only for ACEi combined with ARB [215]. 

Also, the issue of the potential benefit in advanced stages of CKD remains a matter of debate. A number of studies suggested a benefit even at this stage [197,199,208], but an observational analysis pointed to an improvement of kidney function in a number of patients with advanced stage of CKD when RAAS inhibition was arrested [216], which is consistent with the abrogation of the known action of these drugs to decrease glomerular hyperfiltration. This resulted in the advice by some guidance bodies that the withdrawal of these drugs in the late phases of CKD might be considered, when there are no other hard indications for these agents (such as heart failure), especially in patients with renovascular disease (excluded from most RCTs) or when discontinuation of the drug may enable the start of renal replacement therapy to be postponed or avoided [171].

ACEi and ARB have also been repeatedly subjected to health economic analysis and a large number of studies point to the cost effectiveness in both diabetic and non-diabetic CKD [217,218,219,220]. However, some studies point to an initial phase of higher cost, possibly due to the investment in screening and treatment, whereas the benefit by reduction of complications comes only later [217,220].

We conclude that thanks to a large interest of pharmaceutical industry, resulting in a host of well-designed controlled studies, there is not much debate that ACEi and ARB in CKD have a beneficial impact on the progression of kidney disease and probably also on mortality and cardio-vascular outcomes (Table 4, part A). They also definitely offer health economic advantages (Table 4, part B). Especially in diabetic CKD patients, patients with heart failure, and elderly, care should be taken not to apply renin-angiotensin blockers above the maximally tolerated dose (e.g., with regards to the risk of deleterious hypotension). There is a debate on the combined use of both ACEi and ARB together and their unlimited application in advanced CKD [171], and if applied, they should be used with care and with the higher risk of hyperkalemia and acute kidney injury in mind. In view of the complex patho-physiology of many causes of CKD and its progression, and of CKD itself, in most cases, a multi-tiered approach is needed, including not only ACEi or ARB but also other antihypertensive drugs and lifestyle measures [221].

#### 4.1.3. Mineralocorticoid Receptor Antagonists

Previous studies not necessarily focusing on kidney function have shown a positive impact of mineralocorticoid receptor antagonists such as spironolactone on morbidity and mortality of patients with heart failure [222] and as an add-on drug for resistant hypertension [223,224].

Subsequently, the question was raised in how far spironolactone also was a feasible add-on therapy in CKD. In non-diabetic patients with eGFR 30–89 mL/min/1.73 m^2^, adding this drug to a standard RAS inhibitor regime caused less than 1% of serious hyperkalemia and less than 3% of worsening renal function (defined as an eGFR decline by 25–29%) [225]. In an RCT in CKD patients already on ACEi or ARB, add-on spironolactone decreased proteinuria whereas in the control group remaining on the baseline treatment, there was no change [226]. Although eGFR dropped during the first month in the intervention group, after that, it remained stable in contrast to a steady decline in the placebo group. In the spironolactone group, there was a significant rise in serum potassium, especially among patients with more severe CKD (eGFR < 60) [226]. Also, finerenone decreased albuminuria in two studies, the first in patients with diabetic nephropathy and eGFR < 60 mL/min/1.73 m^2^ [227], the second in diabetic nephropathy irrespective of GFR [228]. In the study assessing patients with eGFR < 60 mL/min/1.73 m^2^, 1.5% of all included patients (all on finerenone) developed hyperkalemia, defined as serum K^+^ > 5.6 mmol/L [227]. Maximum 3.2% of cases (in the subgroup on 15 mg/d finerenone) developed severe hyperkalemia necessitating drug discontinuation while in no case eGFR declined with more than the safety threshold of 30% [227]. No hyperkalemia was observed in the second study with no pre-defined upper threshold for eGFR [228].

A few meta-analyses focused on the impact of mineralocorticoid receptor antagonists on specific outcomes. In one such analysis considering both spironolactone and eplerenone, mineralocorticoid antagonism in CKD was shown to reduce blood pressure and urinary protein or albumin excretion, but at the expense of hyperkalemia [229]. Two other meta-analyses, on the same two drugs, came to similar conclusions and found lowering of blood pressure and/or albuminuria/proteinuria, but at the expense of hyperkalemia and gynecomastia (the latter only with spironolactone) [230,231]. A recent subanalysis of a systematic review considering the type of combined RAAS inhibition, already mentioned above, showed a benefit on cardio-vascular mortality with the combination of ACEi or ARB and aldosterone blockade [215]. The effect on glomerular filtration rate was inconsistent [230]. One meta-analysis only focused on the risk for hyperkalemia in ESKD patients in hemodialysis and found no difference [232]. To the best of our knowledge, only one RCT assessed the effect of spironolactone on cardio-vascular and cerebrovascular morbidity and mortality, and this study was undertaken in hemodialysis patients [233]. The primary outcome, a composite of cardio-vascular death or hospitalization, was significantly decreased in the intervention arm, as was the secondary outcome, death of all causes [233].

Summarizing, add-on mineralocorticoid receptor antagonists in CKD definitely have a positive impact on blood pressure and albuminuria, and possibly also on the progression of CKD (Table 4, part B), but results of currently running hard endpoint outcome studies are definitely needed to make firmer conclusions. However, the increased risk of hyperkalemia (see below, section on hyperkalemia), gynecomastia (essentially for spironolactone), and in some studies, an initial decline of eGFR, are drawbacks [234]. Data in ESKD patients in one study seem to point to a positive cardio-vascular impact without definite hyperkalemia, possibly as dialysis offers an additional possibility to kidney function to remove retained potassium, but this single study needs confirmation. In general, studies in this area are also of too limited extent with regards to the length of follow-up and/or patient number.

#### 4.1.4. Hyperkalemia

Hyperkalemia in RAAS inhibition (ACEi, ARB, as well as mineralocorticoid receptor antagonists alone and even more in combination) is more frequent in CKD vs. no CKD although the odds of death are increased to a lower extent at the more severe stages of CKD vs. no CKD [235]. Subsequently, the medical community started considering interventions decreasing serum potassium on a chronic basis in CKD patients on these treatments and who are at risk for or suffer from hyperkalemia, as the only alternative that can be offered is not administering such drugs or lowering their efficiency by decreasing dose.

Next to restriction of dietary potassium intake, the traditional approach to prevent hyperkalemia is pharmacological and based on the administration of the intestinal sorbent polystyrene sulfonate (kayexalate, resonium, either as Na^+^ or a Ca^++^ salt) but recently, a number of alternative sorbents have been developed. In an RCT with patients on RAAS inhibition, one such sorbent (patiromer) induced not only a substantial decrease in serum potassium [236], but also in aldosterone levels [237]. Another sorbent, sodium zirconium cyclosilicate, was tested in patients with hyperkalemia and also generated a decrease in serum potassium [238]. Of note, in both studies, the novel agent was compared to placebo and not head-to-head to polystyrene. To the best of our knowledge, there are also no hard outcome data available, nor analyses of the health economic impact.

The new sorbents have been suggested to be a valuable alternative to polystyrene sulfonate, especially because the latter has been suggested to be relatively ineffective [239], but in the only RCT comparing polystyrene to placebo over a seven day follow-up period, a similar potassium decreasing effect was observed as with the new agents [240]. Another major concern is the risk of severe gastro-intestinal complications such as intestinal necrosis [241], probably rather attributable to the add-on of sorbitol to prevent constipation than to polystyrene per se. However, in one retrospective analysis of 2194 patients receiving the sorbent vs. 121,197 not receiving it, intestinal necrosis was not significantly more frequent in the group on polystyrene [242]. Of note, gastro-intestinal complaints are present as well with the use of patiromer or zirconium cyclosilicate [236,238]. Finally, unspecific cation adsorption (e.g., of magnesium) occurs with polystyrene but also with patiromer [236,241].

Thus, although the new sorbents may offer added value to our current therapeutic arsenal to prevent cardio-vascular complications and progression in CKD, it is difficult to draw any firm conclusions in viewing the lack of hard outcome head-to-head comparisons with polystyrene sulfonate. In addition, the degree of hyperkalemia on its own is claimed to be a poor predictor of cardiotoxicity [125], since other factors such as the speed of development and the clinical condition of the affected patients (comorbidities, concomitant drug intake) are also important determinants of outcomes [125]. 

#### 4.1.5. Beta-Blockade

CKD is frequently associated with sympathetic hyperactivity which might affect blood pressure but also prognosis at large [243,244], and this offers a rationale for beta-blockade to provide additional cardio-vascular protection in renal failure patients [245]. Yet, beta-blocker usage is underexploited in CKD and ESKD, and among hemodialysis patients with sudden death, only 40% received a beta-blocker, while this proportion was only 31% in those dying in the first 12 h post-dialysis, substantially lower than in patients experiencing sudden death in the last 12 h of the interdialytic interval (59%) [246].

The unsatisfactory application of beta-blockade may be due to earlier studies pointing to a less efficient antihypertensive and nephroprotective effect as compared to other antihypertensive agents such as ACE inhibitors [247,248,249] and to data indicating that with some of these beta blocking agents like atenolol the antihypertensive effect does not last for the full 24 h after intake [250]. An impacting factor associated with higher mortality, at least in hemodialysis patients, may be lower cardioselectivity, although the latter data were obtained by retrospective analysis [251]. Also, high dialysability in hemodialysis patients might imply an outcome disadvantage [252]. In addition, beta blockers are a potential source of hyperkalemia [253].

However, the newer vasodilating beta-blockers, such as carvedilol and nebivolol, do not share the same properties as the older drugs in this class [171,245]. In an RCT, carvedilol, as compared to metoprolol, did not affect glycemic control and improved components of the metabolic syndrome [254]. Several RCTs in patients with heart failure and on dialysis [255] or heart failure with CKD not on dialysis [256,257,258] showed a survival advantage with the second or third generation beta-blockers (carvedilol, bisoprolol or nebivolol) vs. placebo. A systematic review considering 8 studies revealed a positive effect of beta-blockade on overall mortality and cardio-vascular mortality as compared to placebo in patients with chronic heart failure and CKD stage G 3 or above [259], but there were insufficient data available to make sound conclusions in patients without heart failure [259]. 

Thus, especially in CKD with heart failure, the newer vasodilating beta-blockers seem to offer added value to other antihypertensives, especially RAAS inhibitors, with regards to overall or cardio-vascular outcomes (Table 4, part B).

### 4.2. Interventions Other than Antihypertensive Treatment

#### 4.2.1. Glycemia Control

a. Intensive Glycemia Control

In the randomized controlled Diabetes Control and Complications Trial (DCCT), intensive glycemia control in type 1 diabetic patients with no or mild retinopathy, lowered the number of patients developing microalbuminuria and albuminuria, however at the expense of a two to threefold increase in hypoglycemic episodes [260]. In this study mean creatinine clearance of the enrolled patients was more than 120 mL/min/1.73 m^2^ and prevalence of albuminuria was low, indicating that the majority of patients had no or early stage CKD, and making generalizability of the results uncertain [260]. A follow-up study showed a persistent positive impact of intensive treatment on surrogate endpoints such as microalbuminuria, clinical albuminuria, the rise in serum creatinine and hypertension [261]. Also in the UK Prospective Diabetes Study (UKPDS), intensive treatment decreased progression of microalbuminuria and for a follow-up of up to 12 years, doubling of serum creatinine [262], together with a decrease in microvascular endpoints, again however at the expense of more hypoglycemia. In this study, mean serum creatinine at the start was in the range of 0.90 mg/dL and prevalence of microabluminuria 11–13%, again pointing to very few CKD cases at enrollment [262]. Likewise, the Action in Diabetes and Vascular Disease: Preterax and Diamicron MR Controlled Evaluation (ADVANCE) trial showed that more intensive glycemia control in type 2 diabetic patients resulted in a lower incidence of nephropathy [263] and a post-hoc analysis of the same study also in the development of ESKD [264]. Like for the DCCT trial here also an analysis during post-trial follow-up showed a sustained benefit in preventing ESKD [265]. In this study ±45% of patients had CKD, 19% had CKD stage G 3, and median eGFR was about 75 mL/min/1.73 m^2^ [264]. Of note, the ADVANCE trial has shown no benefit for intensive glycemic control on mortality [263].

However, in a subanalysis of the Action to Control Cardiovascular Risk in Diabetes (ACCORD) trial [266] which, in contrast to the two previous studies, demonstrated higher mortality with intensive treatment, it was shown that this higher mortality could essentially be attributed to the subgroup with CKD [267]. Controlling hyperglycemia indeed is difficult in renal failure because of higher risk of hypoglycemia as compared to the diabetic population without CKD [268]. Target HbA1C levels should thus account for additional risk factors, aiming at relatively strict control in those with low risk, but with more leniency in those with comorbidities and high risk for hypoglycaemia [269,270].

Of note, many of the data in this section refer to therapeutic regimes that may become less relevant as novel drugs for glycemia control become available, especially for type 2 diabetes (see below, next two sections).

b. SGLT-2 Inhibition

The novel group of glycemia lowering agents, the sodium-glucose cotransporter 2 (SGLT-2) inhibitors, has important hemodynamic and metabolic effects with the potential of cardio-vascular and renal protection [271,272,273]. They have been subjected to several studies on their effect not only on cardio-vascular outcomes but also on kidney function in type 2 diabetes. As compared to placebo, empagliflozin administered in type 2 diabetics with high cardio-vascular risk improved cardio-vascular outcomes and mortality [274], and it also slowed down eGFR loss in post hoc analysis [275]. Similarly, canagliflozin administered to a group of type 2 diabetes patients with high cardio-vascular risk, showed a decrease of cardio-vascular disease and a reduction in progression of albuminuria and loss of eGFR, but no difference in all-cause mortality [276]. There was however also an increase of amputations [276]. Also in a post hoc analysis of a comparison with glimepiride, a nephroprotective effect was described for canagliflozin [277]. Of note, in some of these studies, the moderate (as compared to placebo) but still existent decline in average eGFR for the group treated by empagliflozin was completely reversed to baseline after drug discontinuation (median interval of 34 days), in contrast to placebo [275]. Finally, in a short-term study over 12 weeks, albuminuria also declined vs. placebo with dapagliflozin, another SLG-2 inhibitor [278]. This was however accompanied by a decrease of eGFR vs. placebo, but here also the latter was readily reversible after the drug was arrested [278]. Of note, although none of these studies specifically aimed at CKD, a substantial number of enrolled patients in some of these studies (up to 52%) had an eGFR < 60 mL/min/1.73 m^2^ and/or albuminuria [274,275]. Empagliflozin also reduced urinary albumin/creatinine ratio consistently over a median follow-up of 3.1 years, irrespective of the degree of albuminuria at enrollment [279]. Of note, renal outcomes in most of these studies were secondary endpoints, so that dedicated renal outcome studies are needed for confirmation and those are ongoing [280].

A meta-analysis on various SGLT-2 inhibitors in diabetes type 2 not necessarily with CKD showed a benefit on various cardio-vascular events and mortality, with the exception of a negative effect for non-fatal stroke [281]. 

In an additional analysis of the EMPA-REG OUTCOME, focusing on high-risk patients with cardio-vascular disease and an eGFR < 60 mL/min, empagliflozin improved clinical outcomes, including all-cause mortality [282].

Potential drawbacks are the less potent glucose lowering impact as kidney failure progresses [272] and an increased risk for an unspecific type of ketoacidosis, the so-called euglycemic diabetic ketoacidosis, a type of ketoacidosis with uncharacteristically mild to moderate elevations of glycemia [283]. However, part of these ketoacidosis cases may have been patients with type 1 diabetes using the drug off label. Of note, the incidence of ketoacidosis was low in the EMPA-REG trial [274,275], but this observation was made in the context of an RCT, where monitoring and patient selection are always more strict and side-effects lower than in real life. In addition, it is recommended to avoid these drugs at an eGFR below ≤ 45 mL/min/1.72 m^2^ or even ≤ 60 mL/min/1.72 m^2^ (dapagliflozin) [270], although in some of the above studies, patients with an eGFR below those thresholds were included. Caution has been advised because of the possible increased risk of loss of eGFR (potentially reversible after the drug arrest in a number of cases) or acute kidney injury especially in patients with reduced eGFR at baseline [284]. Finally, possibly related to the enhanced urinary excretion of glucose, there was an increase in urinary and genital infection [274,275,281]. Two studies with canagliflozin showed, essentially in older patients (mean eGFR 75–80 mL/min/1.72 m^2^), bone loss and increased bone fracture rate, which could be a matter of concern in CKD [285,286]. In the EMPA-REG study, however, no increase in bone fracture rate was found [274,275]. Also a systematic review, including several SGLT-2 inhibitors, and mainly driven by the results of the EMPA-REG study, did not mention a negative impact on bone fracture rate [281].

c. DDP-4 Inhibitors and GLP-1 RAs

Data on other novel antihyperglycemic drugs are less persuasive. Although a lower occurrence of albuminuria has been described with the dipeptidyl peptidase 4 (DDP-4) inhibitor, saxogliptin, there was no effect on eGFR or other renal outcomes [287]. Likewise, as recently reviewed, all studies with another novel group of diabetic drugs, the glucagon-like peptide-1 receptor agonists (GLP-1 RAs), in patient groups not necessarily suffering from CKD, showed an improvement in albuminuria but not always in other renal function parameters, while results of cardio-vascular outcomes were contradictory [288,289,290]. Of note, the decline in albuminuria went along with an improvement in glycemic control that as such could have explained the findings [288], so that a potential drug-specific benefit remains speculative. In one study, including patients with cardio-vascular disease and CKD and comparing semaglutide vs. placebo, the drug exerted beneficial effects on cardio-vascular events and new onset or worsening nephropathy [291], but the latter difference was largely driven by differences in new onset macroalbuminuria [288]. Therefore, in our opinion, data on these two drug types are too limited to allow definitive conclusions.

d. Metformin

Finally, metformin is a highly efficient and inexpensive glycemia regulator with beneficial cardio-vascular impact, for which the previous reluctance against its use because of risk for lactate acidosis in CKD has recently been relaxed [269,292,293]. In an observational comparison between sulfonylurea and metformin treatment in diabetics with eGFR > 60 mL/min/1.73 m^2^, metformin was associated with a lower risk for decline of kidney function or death [294], and other data reveal a reduction in mortality in diabetic patients with eGFR < 60 mL/min/1.73 m^2^ under metformin treatment [295,296].

A systematic review also including study populations with CKD, confirmed a benefit of metformin on overall mortality, also in the subgroup with CKD, be it with less benefit in the group with more severe CKD (eGFR 45–30 mL/min/1.73 m^2^) [297].

Given the data suggesting an antifibrotic effect of metformin in other organs than the kidneys [298,299], one may wonder whether metformin could also be useful in the prevention of CKD, independent of the presence of diabetes [300]. Specifically, in adult polycystic kidney disease (ADPKD), metformin was able to decrease cyst volume in a series of experimental models [301]. This mechanism was later on attributed to the inhibition of the extracellular signal-related kinase (ERK) pathway [300]. Data on the impact of metformin on other causes of CKD than ADPKD or diabetic nephropathy are however to our knowledge lacking as of today.

e. Conclusion

Sharp glycemic control may have a nephroprotective impact, but this effect is abrogated as kidney failure advances by increasing risk for hypoglycemia and serious adverse events (Table 5, part A). The SGLT-2 inhibitors are a promising addition to our therapeutic arsenal especially for moderate CKD (Table 5, part A) and may offer novel pathways to protect kidneys, which hopefully will be corroborated by ongoing dedicated studies in diabetic and non-diabetic CKD assessing long-term and hard outcomes, including evolution to ESKD [280], next to socio-economic impact. The beneficial potential of metformin even in advanced diabetic CKD is probably underexploited but also underexplored (Table 5, part A).

#### 4.2.2. Treatment of CKD-MBD

The deleterious impact in CKD of increased phosphate concentrations and disturbances in phosphate homeostasis has been stressed repeatedly [302,303,304], but the evidence about a positive impact by phosphate lowering drug therapy remains more debated [302,305]. In observational analyses, both low and high phosphate levels were linked to increased mortality, with the worst outcomes for serum phosphate > 7.0 mg/dL and no increased risk for values between 3.5 and 6.0 mg/dL [306]. In a meta-analysis, the risk of death increased by 18% for each 1 mg/dL increase of serum phosphate, whereas changes in calcium and parathyroid hormone were not convincingly associated to any outcome differences [307].

a. Phosphate Binders

In observational studies, treatment with phosphate binders of any kind was associated with lower mortality in non-dialysis dependent CKD [308] and hemodialysis [309]. In an RCT, however, phosphate binders of any kind (both calcium and non-calcium), analyzed in an aggregated way, were linked to increased vascular calcification [310]. This effect occurred in spite of a decrease of serum phosphate and parathyroid hormone [310].

Use of calcium containing phosphate binders was not associated with a change of 1-year mortality in an observational study of incident dialysis patients [311]. Titrating the dosage of calcium containing phosphate binders in an RCT, resulting in a group with intensive phosphate lowering, and a group with liberalized phosphate target, generated no differences in major vascular events [312]. A meta-analysis concluded that drug-induced changes in biochemical parameters linked to CKD-MBD could not be associated with any mortality benefit [313]. Likewise, the same authors also could demonstrate no mortality reduction, either with calcium containing phosphate binders vs. placebo [314], or with phosphate binders at large vs. placebo [314], although overall studies were too short to allow enough fatal events to make a difference. 

With the advent of non-calcium containing phosphate binders and accounting for the above-mentioned increase in vascular calcification if phosphate binders were assessed in an aggregated way [310], many studies concentrated on the impact of non-calcium phosphate binders, usually in comparison to the classical calcium-containing binders.

An RCT showed improvement in flow-mediated vasodilatation with sevelamer but not with calcium acetate [315]. Accounting for the current opinion that one of the first changes that occur in the evolution of CKD-MBD is a phosphate-induced increase in FGF-23 [11,316,317], a number of studies assessed the effect of phosphate binders on FGF-23. In an RCT of limited extent in CKD stages 3–4, only sevelamer and not calcium acetate could decrease FGF-23 [318]. Another RCT, comparing sevelamer to placebo, however, showed no impact on FGF-23 or Klotho [319]. Another placebo-controlled study showed no difference with sevelamer in left ventricular mass, systolic and diastolic cardiac function and a difference in FGF-23, only in a subgroup analysis accounting for the compliant subpopulation [320]. Also, with lanthanum treatment, no differences in FGF-23 could be observed within 2 weeks (*n* = 4 per group) [321], but a decrease was reported in a group of 18 patients with a follow-up of 1 month [322]. 

A number of studies assessed hard clinical outcomes with calcium vs. non-calcium containing phosphate binders. In one of the largest controlled trials with prevalent hemodialysis patients, no advantage of sevelamer on all-cause and cause-specific mortality was observed [323]. Secondary endpoint analysis of an RCT comparing sevelamer to calcium binders showed a borderline effect on mortality [324]. Both a smaller and a larger Italian RCT showed lower mortality with sevelamer vs. calcium containing agents in non-dialyzed and dialyzed CKD patients [325,326], although the better outcome in dialysis patients was possibly attributable to better phosphate control [325].

Not surprisingly, the same question was also subjected to many systematic reviews. Four meta-analyses disclosed a lower coronary calcification score with sevelamer vs. calcium-based binders [327,328,329,330]. Whereas early hard outcome analyses were non-conclusive [329] or found an advantage for non-calcium binders which, however, was skewed by heterogeneity [328], more recent analyses observed lower mortality with non-calcium containing binders, especially sevelamer [314,331,332], but the latter agent was also most frequently studied [332]. An editorial comment accompanying one of these meta-analyses stressed the important weight of these findings given the scanty other positive outcome studies in CKD [333]. In one meta-analysis, however, no statistically significant differences were found for mortality, neither for sevelamer nor for lanthanum or iron-based agents, although sevelamer was associated with a 50% lower risk of hospitalization [330]. 

A more or less similar picture emerged from the cost-effectiveness analyses. Whereas the earlier studies pointed to a higher cost per QALY gained especially in hemodialyzed patients [334], more recent analyses favored sevelamer over calcium salts [335,336,337], a trend that is possibly favored by the expiry of the patents of some of the non-calcium binders. One analysis found that lanthanum was more cost-effective than sevelamer [338].

Thus, although some but not all studies support superiority of non-calcium containing phosphate binders over those containing calcium, it remains even more uncertain whether phosphate binders at large are beneficial (Table 5, part B), an analysis possibly skewed by the aggregated consideration of the several types of binders (both calcium containing and non-calcium containing) in the same studies. Thus, recent reviews point to the remaining uncertainty of targeting phosphate in the treatment of CKD [302,305], and also stress the underexploitation of dietary intervention (see above, section on diet) [302,305]. However, it is recommended that calcium-based phosphate binders should be restricted in the case of hypercalcemia, adynamic bone disease, persistently low parathyroid hormone or in the presence of vascular calcification [339].

b. Calcimimetics

Next to phosphorus, calcium, and FGF-23, a fourth player in the CKD-MBD axis is parathyroid hormone (PTH). Calcimimetics were added to our therapeutic arsenal to pursue a medically invoked decrease of parathyroid hormone. Treatment with cinacalcet was associated with a decrease in PTH as well as to an improvement of bone turnover parameters and of bone histomorphometry [340]. An aggregated analysis of four different blinded RCTs with similar protocol structures showed a reduction in the risk of parathyroidectomy, fracture and cardio-vascular hospitalization [341]. In a small RCT on surrogate endpoints, cinacalcet decreased vascular and cardiac valve calcification [342]. However, a large RCT in hemodialysis patients with moderate-to-severe hyperparathyroidism (The EValuation of Cinacalcet HCl Therapy to Lower CardioVascular Events trial—EVOLVE) found no superiority of cinacalcet over placebo regarding death or hard cardio-vascular outcomes [343]. Subsequent secondary analyses could reveal no difference with cinacalcet in fracture rate in unadjusted assessment, which, however, became significant after adjustment for confounders [344]. In addition, another subanalysis showed that cinacalcet lowered FGF-23, and that in the subgroup with lower FGF-23 also cardio-vascular death and major cardio-vascular events were reduced [345]. A meta-analysis revealed no positive impact of cinacalcet on mortality [346]. Cinacalcet is only indicated in dialysis patients. 

The alternative intervention to combat hyperparathyroidism, parathyroidectomy, has rarely been subjected to scrutiny. In a Japanese observational analysis on a large registry of hemodialysis patients, parathyroidectomy was linked to lower cardio-vascular mortality risk, and this association remained significant when subjected to propensity and sensitivity analyses [347]. In this study, conservative treatment to lower PTH consisted of vitamin D receptor activators, and not of cinacalcet [347].

Thus, the impact of reducing PTH, either pharmacologically or surgically remains uncertain (Table 5, part B) as hard outcome benefit remained restricted to observational data or secondary analyses.

c. Neutralization of Fibroblast Growth Factor-23 (FGF-23)

As the rise in FGF-23 precedes the rise of parathyroid hormone and phosphate, the question of whether the increase in FGF-23 is not the primary event in the development of secondary hyperparathyroidism [348] may be raised. However FGF-23 neutralization with antibody in rats with kidney failure improved hyperparathyroidism but increased mortality [349]. For the time being, we are not aware of any clinical study directly assessing FGF-23 inhibition. The currently held patho-physiologic concept attributes an adaptive rather than a primary role to FGF-23. Subsequently, nowadays, the main role of FGF-23 is conceived to protect the body against phosphate retention as GFR decreases, although it should be stressed that this compound has also been shown to alter biological functions [316,350,351].

d. Vitamin D and Analogues

Many patients with CKD have low serum levels of 25(0H)-vitamin D3 (calcidiol) or calcitriol, but to the best of our knowledge, randomized placebo-controlled trials supporting a benefit of their supplementation on hard end-points are lacking. However, two meta-analyses, mainly including studies with active vitamin D compounds or analogues underscored the risk for hypercalcemia, especially in combination with calcium-based phosphate binders [352,353]. In one RCT, paricalcitol supplementation added to RAAS inhibition in diabetic nephropathy reduced albuminuria [354], and the two meta-analyses mentioned above supported this benefit on proteinuria for vitamin D products at large [352,353]. However, these findings remain restricted to albuminuria/proteinuria, and in spite of several data pointing into this direction, in our opinion, confirmation by demonstrating a benefit on more robust parameters of progression of CKD (e.g., the slope of eGFR) and/or hard cardio-vascular outcomes is needed. In an RCT, eGFR declined more quickly with paricalcitol than with placebo, but eGFR at the start was 14% lower in the paricalcitol group [355].

Due to the scarcity of hard evidence, vitamin D derivatives, calcimimetics and the combination of both are recommended at an equal level in dialysis patients as pharmaceutical options for lowering parathyroid hormone in CKD [339].

#### 4.2.3. Treatment of Dyslipidemia

Combating hypercholesterolemia by statin therapy in the non-CKD population prevents cardio-vascular events in subjects with hypercholesterolemia [356] but also in subjects with other risk factors like increased C-reactive protein (CRP), even in the absence of hypercholesterolemia [357]. This benefit has been described for subjects both with and without a previous cardio-vascular event [358].

In the population with CKD, cholesterolemia typically regresses as kidney disease advances, due to malnutrition and inflammation [1]. According to Kidney Disease Improving Global Outcomes (KDIGO) guidelines, identification of dyslipidemia (high total or LDL cholesterol, low HDL cholesterol, high triglycerides) is deemed useful to detect severe hypercholesterolemia and/or hypertriglyceridemia, to identify potential remediable (secondary) causes, and to assess overall cardiovascular risk in patients with non-dialysis CKD aged < 50 years. Unless in situations where results would possibly imply changes in management, follow-up of lipid levels is not recommended by lack of evidence of a benefit [359]. 

Observational analyses had suggested an outcome benefit for statins in patients with moderate CKD with or at risk of coronary disease [360]. The RCT Study of Heart and Renal Protection (SHARP) trial and subsequent meta-analyses showed the usefulness of administering cholesterol lowering agents (statin + ezetemibe) to obtain an absolute reduction of LDL cholesterol in patients with CKD not on dialysis to decrease the associated cardio-vascular risk or overall mortality risk [361,362,363,364,365].

In a subanalysis of the SHARP trial, LDL cholesterol lowering in CKD patients not on dialysis did not impact on the evolution to ESKD or doubling of baseline creatinine [366]. However, in one meta-analysis, statins also reduced proteinuria in patients with CKD but not progression of CKD [362], whereas in another analysis, both proteinuria and eGFR decline per unit of time were altered [361], but again without an impact on the occurrence of kidney function events, defined as a decline in eGFR by 50%, doubling of serum creatinine or ESKD [361].

Cholesterol lowering therapy is recommended by KDIGO in all non-dialyzed CKD patients of more than 50 years old [359]. According to a quality standard issued in 2017 by the British medical guidance body, the National Institute for Health and Care Excellence (NICE), a statin should be offered to all patients with CKD, including those with CKD stages G 1 and 2 [367].

In contrast, this benefit of statins was less convincing in dialyzed populations [363,368,369,370,371], discouraging their initiation in ESKD, irrespective of inflammation or malnutrition, although they can be continued if already started [359]. 

In the non-CKD population, statins have been considered cost-effective in patients with vascular disease [372,373], but also as primary prevention in patients at lower risk [374,375,376].

Although cardio-vascular events incur a substantial increase in expenditures for CKD patients [377], cost-effectiveness data on statins in CKD patients are scarce. Based on a decision-analytic model, statins were cost-effective especially in CKD subjects with higher risk (e.g., hypertension or males above age 65) and if generic statins were used [378]. Based on the SHARP data, the combination of statins plus ezetemibe was especially cost-effective in patients with higher cardio-vascular risk [379], but the authors point out that less costly regimes (e.g., statins alone) are likely to be more cost effective [379]. CKD patients will also take profit of including measures favoring lifestyle modification (see above, section on lifestyle) [380].

Recently, novel strong cholesterol lowering compounds [monoclonal antibodies against proprotein convertase subtilisin/kexin type 9 (PCSK9) or angiopoietin like 3 genes (ANGPTL3)] have been added to our therapeutic arsenal [381,382], but they may be a matter of health-economic concern, especially since excessive costs as compared to the current standard are expected [383]. Some recent health-economic analyses on these drugs should be considered with care because of industry involvement in their generation and, as they only consider the non-CKD population, their conclusions can by definition not be extrapolated to the context of CKD [384,385].

In conclusion, there is now a solid underpinning for the therapeutic benefit of statins in non-dialyzed CKD patients at risk and when generic compounds are used, and even for general prevention in all non-dialyzed CKD without accounting for risk or cholesterol levels (Table 5, part C). It is at present, impossible to define the place of the novel anti-cholesterol agents in CKD by lack of data, but the cost issue may impose substantial obstacles against their implementation in unselected cases with kidney disease. Shared decision making is highly relevant for installing cholesterol lowering therapy at large but is especially relevant for the more resource consuming options, where costs are immediate, and the presumed benefits are projected in future [386].

#### 4.2.4. Anti-Inflammatory Therapies

Inflammation plays a fundamental role in the cardio-vascular complications associated with CKD, with as key elements the activation of the redox-sensitive nuclear transcription factor kappa B (NF-κB) and the subsequent release of several cytokines and chemokines, including a number of interleukins (ILs) such as IL-6 and monocyte chemoattractant protein-1 (MCP-1), also named C-C motif ligand 2 (CCL2) [1].

In spite of some experimental studies and clinical trials in the non-CKD population suggesting that interfering with those systems may have a beneficial impact on cardio-vascular outcomes [387,388], these interventions also increase the risk of infection. Unspecific approaches reducing inflammation (e.g., statins) were up until now not that successful in CKD to produce health benefits. In the “An Assessment of Survival and Cardio-vascular Events” trial (AURORA), rosuvastatin lowered (−27%) serum CRP, but failed to reduce mortality or the risk for cardio-vascular events [368]. Resistance to interventions targeting inflammation may depend on the severity of inflammation in ESKD (in AURORA median CRP in the active arm remained 4 times above upper normal), and the multifactorial origin of the vascular disease in CKD and ESKD.

a. NF-κB Inhibition

Interfering with pro-inflammatory and oxidative mechanisms by reducing NF-κB activation and at the same time activating nuclear factor erythroid 2-related factor 2 (Nrf2), seemed an attractive option to reduce the high risk for all-cause and cardio-vascular death in advanced CKD. A limited phase 2 study testing the NF-κB blocker/Nrf2 activator, bardoxolone, had demonstrated an increase of eGFR in diabetics with category G3b-G4 CKD [389]. However, a more extended trial testing bardoxolone in G4 CKD diabetics was terminated prematurely because of excessive cardio-vascular death risk related to early volume overload in the bardoxolone arm [390]. Yet, the conditions that made the drug fail in a number of patients might help to find specific populations in which this therapy may still be useful [391]. After reportedly presenting novel promising results of a pilot study, the drug is currently tested in phase 3 for Alport disease [392,393]. Although the importance of testing drugs for orphan diseases should be acknowledged, care should be taken with the interpretation of eventual results because of the inherent size and time limitations of the novel studies, the previous dismal outcomes, and the risk for off label use if a positive result would be obtained with the novel study [394].

b. CCR2 Inhibition

Aiming at a different target in the inflammatory chain, a CCR2 inhibitor had a positive effect on albuminuria in diabetes type 2 as an add-on to RAS inhibition, but results were not entirely consistent, as a maximum impact was reached at week 12, with an almost entire disappearance of the bonus at week 56 with the highest dose of the drug [395]. No effect was seen on eGFR [395]. In a comment, the therapy was called promising as an add-on to ACEi or ARB, however, leaving many remaining questions about the true patho-physiology of diabetic nephropathy unanswered [396]. Emapticap pegol (NOX-E36), a Spiegelmer targeting the CCR2 ligand CCL2, also reduced albuminuria at week 12 in an exploratory study, but no significant difference vs. the placebo treated control group was found [397].

c. Pentoxifylline

In an observational cohort study in Taiwan, on non-dialyzed CKD patients, pentoxifylline in combination with RAS inhibition lowered the risk of the composite of long-term dialysis or death [398]. In addition, in a small RCT assessing the effect of the add-on of pentoxifylline to RAS inhibition, not only albuminuria and urinary tumor necrosis factor (TNF)-α but also the decline in eGFR was attenuated in patients with diabetic nephropathy [399]. A recent meta-analysis of RCTs confirmed this benefit for pentoxifylline combined to RAS inhibition [400].

Although these data are of a fairly limited extent and would need confirmation in larger scale studies not based on composite endpoints, pentoxifylline, especially if added to RAS inhibition, may play a role in protection against mortality and/or progression of CKD. 

d. Combating Periodontal Disease

Periodontal disease is a major cause of inflammation [401] which has been associated with cardio-vascular disease [402]. An RCT was started in 2015 in aboriginal Australians to check the impact of periodontal care in CKD [403].

e. Conclusion

Overall, data on specifically reducing inflammation in CKD are not robust enough to definitely favor any directed approach, with the possible exception of pentoxifylline (Table 5, part D). 

#### 4.2.5. Correction of Hypomagnesemia

Although magnesium ingestion in part is dependent on dietary intake, the main option to increase its concentration is by oral supplementation in pharmacological doses, or, in dialysis patients by modifying dialysate magnesium content [404].

There is quite extensive experimental evidence that magnesium has a positive impact on factors linked to cardio-vascular damage [405]. Most relevant in this discussion might be the property of low magnesium to induce osteogenic transformation in the vessel wall, especially in vascular smooth muscle cells, and the capacity of magnesium addition to reverse this effect [406,407,408].

In 1987, an observational link was already described in ESKD between higher circulating magnesium and a lower number of arterial calcifications [409] and in a small RCT in patients with coronary artery disease with unspecified kidney function, and excluding patients with a serum creatinine > 3 mg/dL, oral magnesium supplementation was beneficial for endothelial function [410]. 

The question arises what the effect of such an intervention would be in CKD. An RCT assessed the phosphate binding effect and tolerability of a magnesium salt in hemodialysis patients and found a similar phosphate lowering effect as with sevelamer hydrochloride without major adverse events [411]. The intervention implied the administration of 60 mg of elemental magnesium and caused a significant increase of serum magnesium by 0.3 mmol/L. Parathyroid hormone decreased significantly at 25 weeks [411]. No hard outcomes were studied [411]. Small randomized trials focusing on surrogate outcomes point to a decrease of carotid intima media thickness [412,413] and serum calcification propensity [414]. In addition, in an RCT, a borderline significant improvement of arterial calcification has been described in hemodialysis patients receiving magnesium supplementation [415]. In a recent meta-analysis, high magnesium intake was linked to a lower risk of hypertension [416].

Associations with hard outcomes were up till now only obtained from observational analyses. Higher serum magnesium was shown to attenuate the link between serum phosphate and progression of CKD [417]. In several analyses, hypomagnesemia was associated with an enhanced risk of overall and cardio-vascular death [418,419,420]. Mild hypermagnesemia was in hemodialysis patients connected to a lower risk of hip fractures [421] and less overall and cardio-vascular mortality [422].

Overall, surrogate outcome and observational data are suggestive of a link between hypomagnesemia and enhanced cardio-vascular and overall mortality, but interventional data are less convincing (Table 5, part E) [404,405,423]. There is an obvious lack of randomized trials assessing hard outcome impact of magnesium supplementation in CKD [405] and the availability of magnesium containing phosphate binders may be of help to overcome this problem [423] if these increase serum magnesium, as suggested before [411]. However, the difficulties encountered to increase serum magnesium by oral supplements may be an obstacle to reach this purpose [424], especially in patients with no or moderate CKD.

#### 4.2.6. Correction of Metabolic Acidosis

In experimental studies, acidosis has been associated with muscular proteolysis and negative nitrogen balance, which are metabolic steps involved in muscle and protein energy wasting [425].

Randomized controlled trials on acidosis correction were mostly small and focused on surrogate outcomes. An RCT found no difference in serum albumin and total lymphocyte count as nutritional indicators [426]. In another RCT, however, bicarbonate supplementation was linked to an improvement of nutritional parameters (protein intake, mid-arm muscle circumference, serum albumin) and, in addition, to a slower progression of CKD [427]. This study found no major adverse events and no difference in blood pressure in spite of higher sodium intake with the bicarbonate regimen [427]. There was also a decrease of serum potassium in the intervention arm [427]. A meta-analysis found 6 studies (4 RCTs), which as a whole, suggested slower progression of CKD with alkali therapy, but differences in study protocols and small sample sizes precluded definitive conclusions [428]. 

Several observational studies assessed the relationship between plasma bicarbonate and mortality. For patients who were not yet on dialysis, the lowest mortality was found in the group with serum bicarbonate 26–29 mmol/L and higher mortality was observed both with lower and higher bicarbonate levels [429]. In a large Japanese study, neither low nor high adjusted pre-dialysis serum bicarbonate values were linked to increased mortality, and the only acid-base parameter related to mortality was a pre-dialysis pH ≥ 7.40 [430]. Finally, in one of the DOPPS analyses, hemodialysis patients with a moderate acidosis (21.1–22.0 mmol/L) showed the best outcomes and both mortality and hospitalization rates were higher in patients with higher and lower bicarbonate values [431]. At least two of the studies showed a J-shaped curve [429,431]. In another DOPPS analysis, high dialysate bicarbonate levels, likely contributing to postdialysis alkalosis, were associated with higher mortality [432]. 

A potential concern of persistent mild alkalosis is the increased risk of vascular calcification, and this should be addressed by future alkalinization trials [433]. In a clinical trial observing increased vascular calcification for patients on diverse phosphate binders, all the binders tested contained carbonate or acetate, which all have the potential to increase serum bicarbonate [310]. 

In general, the results on acidosis and its correction are conflicting (Table 5, part F). There may be a positive impact of correcting acidosis on the progression of CKD, but the study design and their limited number do not allow firm conclusions. Observational data on mortality are contradictory. A negative impact, if any, seems to be present both at lower and higher bicarbonate levels. This may be linked to the different factors impacting acid-base balance and its consequences that may cause confounding, e.g., malnutrition driven by low protein intake may increase bicarbonate levels, whereas acidosis may promote protein breakdown and malnutrition.

#### 4.2.7. Aryl Hydrocarbon Receptor (AhR) Blockade

The aryl hydrocarbon receptor has traditionally been considered as a molecule mediating toxicity, but more recently a role in immune function and carcinogenicity have been recognized [434,435]. Subsequently, a procoagulant role via the activation of tissue factor was also demonstrated [436].

A patho-physiologic role of AhR in the uremic syndrome was demonstrated first in 2010 by showing activation in the presence of the indole, indoxyl sulfate [437]. This activation has pleiotropic effects and plays a role in endothelial cell senescence [438], vascular inflammation [439] and the list of uremic retention solutes with the potential to activate AhR has been extended beyond indoxyl sulfate to other tryptophan metabolites such as indole acetic acid, indoxyl glucuronic acid and the kynurenines [440,441].

A pro-coagulant role by indoxyl sulfate and later on also the kynurenines via AhR activation and increased tissue factor expression has been suggested [436,442,443] and could be blocked in vitro by various AhR antagonists [442,444].

These findings open new avenues for pharmacologic intervention to antagonize the action of uremic toxicity [444]. Salicylamide, but not acetylsalicylic acid, as well as several specifically developed AhR antagonists were shown to block AhR activity [442,445]. To the best of our knowledge, there are no human interventional trials with AhR antagonism. Of note, several natural nutritional components, such as flavonoids, are claimed to antagonize AhR [435]. However, up till now, most in vivo work on AhR blockade has been done in animal experiments [446,447].

Overall, AhR antagonism seems like a promising pharmaceutical option for developing future therapies directly countering pharmaceutically the clinical consequences of uremic toxin retention. However, for the time being, we are not aware of any clinical tests of AhR antagonists both in human non-uremic or uremic conditions. Possibly, the ubiquitous presence of AhR receptors precludes their use for specific indications, and more specific drugs may be needed if, e.g., specifically neutralizing pro-coagulatory effects [434,435].

#### 4.2.8. Anticoagulation

The population with CKD is at high risk of thromboembolic events, either as such, or linked to atrial fibrillation, one of the most frequent cardio-vascular complications [448], and this offers a rationale for anticoagulant prevention. However, CKD and especially ESKD patients are also prone to bleeding, a tendency that is further enhanced by anticoagulation and the reduced clearance of anticoagulant drugs and their metabolites in the presence of kidney failure.

In diabetic patients under dual antiplatelet therapy, those with CKD showed higher platelet reactivity vs. non-CKD patients and thus a blunted antiplatelet response [449]. In patients receiving clopidogrel and undergoing percutaneous coronary intervention, those with eGFR < 60 mL/min/1.73 m^2^ had more adverse events (both ischemic and bleeding events) compared to those with higher eGFR [450]. In the same vein, treatment with clopidogrel for 1 year as compared to one month after percutaneous coronary intervention, resulted in a decrease of cardiovascular events in those treated for 1 year and with eGFR above 60 mL/min/1.73 m^2^ but not in those with lower eGFR. In addition, patients on clopidogrel experienced a higher bleeding risk, but this effect was independent of kidney function [451]. In a DOPPS analysis, considering only patients on hemodialysis, aspirin treatment was associated with less stroke but more myocardial infarctions and overall cardiac events than no aspirin [452]. The latter study, however, had a high potential for bias by indication. 

Perhaps the type of anticoagulant plays a role. In an RCT, ticagrelor was superior to clopidogrel in inducing an antiplatelet effect [453], but the study assessed no hard outcomes and was underpowered to compare bleeding tendency.

In a meta-analysis, antiplatelet agents in adults with CKD prevented myocardial infarction, and had uncertain effects on mortality and increased bleeding, but heterogeneity in study design made the interpretation difficult [454].

In older CKD patients with atrial fibrillation, anticoagulant prescription (mainly vitamin K antagonists) did not decrease the risk of stroke but increased bleeding events and mortality as compared to those not anticoagulated [455]. Likewise, warfarin in hemodialysis patients with atrial fibrillation was associated with higher stroke risk (both hemorrhagic and ischemic) and no difference in overall mortality and hospitalization rate [456]. However, in another larger observational study, warfarin in patients with atrial fibrillation showed a decrease in cardio-vascular events in CKD patients, especially in those with a high risk score [457]. 

Most reviews agree that data on anticoagulant and antiaggregation use in CKD are conflicting with regards to cardio-vascular and overall mortality (Table 5, part G) and that potential benefits come at the expense of a higher bleeding risk [448,458,459,460,461]. As most available data is based on observational studies, an urgent need for well-designed RCTs has been stressed [460].

The recent introduction of several novel or non-vitamin K oral anticoagulants (NOACs), also named direct oral anticoagulants (DOACs), opened a new therapeutic pathway. In spite of data suggestive of their usefulness vs. classical warfarin in the general population [462], to the best of our knowledge, insufficient information is available about their application and the health-economic impact in CKD. In addition, they should be used with care in CKD, as their renal elimination may be hampered progressively as renal function deteriorates [463,464].

However, the possible benefit of NOAC vs. warfarin on CKD progression deserves attention. It has been reported that oral anticoagulation with vitamin K antagonists may be associated with hematuria-related AKI during episodes of overanticoagulation, giving rise to the so-called warfarin-related nephropathy [465]. While this condition may be associated with other anticoagulants as well [466], more stable anticoagulation or other actions of NOACs may underlie the recently described lower risk of adverse renal outcomes (AKI, decrease of eGFR) with NOACs, particularly dabigatran and rivaroxaban, than with warfarin, in several observational studies [467,468,469,470]. However, in a meta-analysis of 10 RCTs (4 on NOAC vs. vitamin K antagonists), NOACs showed a similar risk of renal failure as the other anticoagulants, including warfarin [471]. However, most data in this analysis had been retrieved from public assessment reports, and the definitions of renal failure were highly variable [471]. 

#### 4.2.9. Prevention of Kidney Fibrosis

Kidney fibrosis is a key element in the progression of renal failure [472,473,474]. The process is influenced by many other factors at play in CKD such as inflammation, hemodynamic conditions and glucose metabolism [472]. In this publication, many of the interventions with the potential to impact on kidney fibrosis have been discussed in the specific sections corresponding to those mechanisms.

The key cytokine in fibrosis is transforming growth factor-β (TGF-β) [473,474]. In spite of promising experimental data with TGF-β antagonism [475], only a few clinical outcome data support such approach to prevent progression of CKD. In an open label longitudinal study on 18 patients, pirfenidone, a drug used to treat idiopathic pulmonary fibrosis, reduced the slope of eGFR decline by 25% in focal segmental glomerulosclerosis, without inducing a change in blood pressure or proteinuria, but assessment of histological differences was lacking [476]. In a small randomized trial (25–26 patients per arm), pirfenidone emanated in a better evolution of eGFR in the 1200 mg/day arm, but with a high drop-out rate and no difference in eGFR in the 2400 mg/day arm [477].

Most successes in this area have been obtained with anti-inflammatory, metabolic and hemodynamic agents (see above, sections on antihypertensives, glycemia control and anti-inflammatory therapies). Likewise, there are also other studies on similar agents in progress or of which the results have not yet been published in peer reviewed journals, which have the potential to further broaden our therapeutic insight in this issue [472].

Another regulator of fibrosis is bone morphogenetic protein-7 (BMP-7) [478], a compound that has been used locally to stimulate bone growth in the past, e.g., in fractures, but influencing the activity of this factor may be even more remote from the clinical application in CKD than TGF-β antagonism [479].

In general, data on direct inhibition of fibrosis is too scanty to allow definite conclusions (Table 5, part H).

#### 4.2.10. AST-120 (Kremezin^R^)

AST-120 is a carbonaceous oral sorbent that in animal experiments had been shown to adsorb indoxyl sulfate and probably other organic uremic retention solutes as well [480,481], and which is currently available for therapeutic use only in some Asian countries [482]. In experimental studies, indoxyl sulfate had been linked to kidney fibrosis and progression of kidney failure [483], which was the rationale for introducing AST-120 into the (essentially Asian) market as a nephroprotective agent.

Subsequently, AST-120 was applied in a number of small randomized controlled human studies in Asia on kidney failure progression which showed a favorable renal outcome with this compound vs. placebo [484]. In type 2 diabetics, early initiation of AST-120 stunned progression of CKD [485]. In patients with moderately severe CKD with and without diabetes, a beneficial effect of the sorbent on the decline of eGFR was shown, which was, however, only a secondary endpoint of the study [486]. In non-diabetic CKD patients, the sorbent was associated with a reduction of proteinuria, a less pronounced rise in serum creatinine, and less tubular damage, together with a decrease of parameters of inflammation and oxidative stress [487]. In an RCT, AST-120 had a beneficial effect on serum creatinine together with an increase of the sensitivity to erythropoietin (EPO) as illustrated by higher hemoglobin levels in spite of similar EPO doses [488]. AST-120 was also shown to be cost-effective when treating type 2 diabetics with advanced kidney disease [489].

However, a large RCT ran in Europe, and the USA could show no benefit on the evolution of kidney function as the primary outcome [490]. In this study, the evolution of the concentration of uremic toxins such as indoxyl sulfate was not checked, whereas adherence to therapy was controlled only by assessing the returned medication [490]. A subanalysis in the patients enrolled in the study in the USA showed a delay in the treated group for reaching the primary endpoint which was a composite of RRT and doubling of serum creatinine [491], and this evolution was essentially observed in the group with diabetic nephropathy [491]. A further post hoc analysis showed that the benefit of AST-120 was limited to patients on ACEi/ARB with hematuria and urinary protein/creatinine ratio ≥ 1 [492], although this type of post hoc subgroup analysis is prone to substantial bias, especially as hematuria, as one of the parameters accounted for, is far from specific for intrinsic kidney disease.

Another RCT considering AST-120 vs. placebo also showed no difference in progression of CKD, but there were also no significant differences in indoxyl sulfate concentration over the 3 year follow-up period [493]. Again, a secondary analysis was subsequently undertaken and showed a number of subgroups where AST-120 was beneficial, namely in the most adherent patients and those with diabetic nephropathy [494]. In addition, the best results were seen in the patients in whom indoxyl sulfate decreased most over time [494]. The same post hoc analysis also showed less cardio-vascular events with AST-120 [494].

A meta-analysis suggested a benefit for AST-120 but also stressed that the evidence available was mainly based on secondary outcomes from low quality studies with small sample sizes [495].

Taken together, the current primary outcome evidence is too weak to support nephroprotective treatment with AST-120 (Table 5, part I) [482], especially with the large RCTs being negative. Most of the benefit was only found at secondary analysis in subpopulations. Whether the lack of primary outcome differences is due to imperfections in the study design, lack of adherence (ingestion of a substantial number of capsules is required), genetic or lifestyle predisposition or a preferred effect in specific subpopulations such as patients with diabetic nephropathy or more severe kidney disease, remains unclear. The post hoc analyses may be helpful in designing new RCTs for better defined target populations.

### 4.3. Targeting Specific Uremic Retention Solutes

The uremic syndrome results in an amalgam of patho-physiologic changes which at least in part can be attributed to biological/biochemical (toxic) actions of the solutes that are retained in the body, due to inadequate removal by the failing kidneys [11]. Although most strategies to decrease the concentration or activity of these solutes are unspecific and extracorporeal, some pharmaceutical approaches are used to impact the concentration or action of specific solutes [11]. Suppression of the action of parathyroid hormone and FGF-23 has been discussed separately in the section on CKD-MBD (see above). Apart from the two solutes mentioned above, we could retrieve only five solutes or groups of solutes that had been submitted to interventional studies to decrease their concentration or activity: the advanced glycation end products (AGEs), the cytokines, endothelin A, homocysteine and uric acid. 

#### 4.3.1. Advanced Glycation End Product (AGE) Reduction

Many interventional attempts have been made to reduce AGE levels by drug treatment but none of the tested options has currently emanated in approved therapies for clinical use [496]. In an RCT, the AGE formation inhibitor pyridoxamine hydrochloride (pyridorin) did not impact the progression of CKD unless in the subgroup with the lowest serum creatinine at baseline [497]. One of the most recent acquisitions is glyoxylase-1 induction, which in pilot studies of obese subjects resulted in the breakdown of methylglyoxal, improved glycemic control, an increase in insulin sensitivity and better vascular function [498,499].

#### 4.3.2. Cytokine Antagonism

Many of the cytokines are specific pro-inflammatory uremic retention solutes, and several studies have assessed strategies to decrease their concentration or activity [500]. 

In a proof of concept study in inflamed hemodialysis patients, those randomized to the IL-1β inhibitor anakinra showed a decrease of CRP and IL-6, whereas there was an increase in the placebo-treated patients [501]. 

In patients with rheumatoid arthritis, anti-tumor necrosis factor-α (anti-TNF-α) therapy decreased cardio-vascular events, myocardial infarction, and stroke according to a meta-analysis of observational and randomized studies that, however, did not consider kidney function [502]. Moreover, this therapy had a beneficial impact on the evolution of GFR in patients with rheumatoid arthritis [503]. In a small RCT in hemodialysis patients, the TNF-α blocker etanercept increased serum albumin and prealbumin as markers of inflammation and nutritional status [504]. However, the increased risk of infection, especially with mycobacterium in a population that is already prone to infectious disease is a drawback of this type of therapies in CKD.

We are not aware of further clinical outcome studies with cytokine antagonists in CKD.

#### 4.3.3. Endothelin A Receptor Blockade

Endothelin has been linked to increases in blood pressure, renal inflammation and vascular damage [11]. In a short term randomized cross-over study in CKD patients, endothelin-A receptor blockade resulted in decreases of plasma uric acid, asymmetric dimethyl arginine (ADMA,) proteinuria, hypertension and vascular stiffness [505,506]. Doubts on the long-term use of these agents were, however, raised when 3 to 6 months of treatment in diabetics and non-diabetics were not only related to a decrease in proteinuria, but also to a rise in fluid retention inducing increased mortality [507,508,509]. Maybe these problems could be overcome in future trials by using lower doses or more selective blockers. Another option could be to define the patient phenotype at risk for complications as well as the potential non-responders and avoiding endothelin receptor antagonists in those populations [510,511,512]. However, for the time being, knowledge is insufficient to defend the systematic use of these drugs in CKD. A phase 3 RCT of endothelin-A receptor blockade in patients with diabetic nephropathy [513] was prematurely stopped in December 2017 as it was unlikely that the expected number of endpoints would be met [514]. 

#### 4.3.4. Homocysteine Lowering

Homocysteine is a cardio-vascular risk factor in the general population and has been shown to play a patho-physiologic role in cardio-vascular damage [11]. Homocysteine levels can be lowered by administration of folic acid, vitamin B6 and/or vitamin B12 [11]. 

In four RCTs in CKD, vascular events or fatal outcomes were not reduced by homocysteine lowering therapies with the administration of folic acid alone or combined to other B vitamins [515,516,517,518]. However, in all but one of these studies, the control patients had been subjected to folic acid fortification in regular food [515,516,517,518].

A subanalysis of a large RCT on the effect of folic acid on stroke in a large population of Chinese hypertensive patients [519] showed in the subgroup with CKD that folic acid combined to enalapril more efficiently refrained progression of CKD than enalapril alone [520]. This study obviated the bias of folic acid fortification in the general population, but on the other hand, was restricted to hypertensive Chinese which might have implied specific genetic and metabolic factors.

Two meta-analyses, one of which being based on observational data [521] suggested a reduction of cardio-vascular disease (but not overall mortality) associated with the application of homocysteine lowering preparations in CKD [521,522]. This effect was seen especially in subpopulations that were not or only partially subjected to folic acid fortification, or that showed a lowering of homocysteine by more than 20% in the treated group, irrespective of folic acid fortification [522]. Many studies in these meta-analyses were small, which might detract from the credibility.

Thus, attempts to study the impact of homocysteine lowering vitamin B preparations in CKD have been subjected to confounding by the presence of folic acid fortification in the general population. Partially due to this, clinical data from individual studies seem too conflicting to offer convincing arguments about the role of homocysteine lowering in CKD. Nevertheless, it is of note that two meta-analyses pointed to a cardio-vascular benefit while the therapeutic option, vitamin administration, is barely harmful.

#### 4.3.5. Uric Acid Lowering

Uric acid has been linked to pro-inflammatory processes, vascular damage, and progression of kidney disease [11]. The concentration can be selectively decreased by specific medication, such as allopurinol, the newer xanthine oxidase inhibitor, febuxotat, or probenecid.

In an observational study of CKD patients, treatment with allopurinol was associated with improved arterial stiffness, even after correction for confounders [523]. In a Japanese section of the DOPPS, allopurinol was associated with overall lower mortality in a subpopulation without previous cardio-vascular events [524]. 

A few RCTs assessing the impact of uric acid lowering drugs on endothelial function showed no positive effects [525,526]. One of these studies was undertaken in CKD patients [525]. On the other hand, in at least one other study of hyperuricemic heart failure patients, allopurinol was shown to improve peripheral vasodilator capacity [527]. In a short-term study, allopurinol was shown to decrease blood pressure in young adolescents with newly diagnosed essential hypertension [528], but according to a recent Cochrane systematic review including more than 20 studies, results were overall negative (apart from borderline significance for clinic systolic blood pressure but no effect on 24 h blood pressure), with low quality studies and small therapeutic effects [529]. In another RCT in CKD, the group treated with allopurinol had a lower number of cardio-vascular events, a lower CRP, fewer hospitalizations and a slower progression of kidney failure, which was confirmed by post hoc long-term analysis [530,531]. However, the small sample size of the study was a limitation, so this needs confirmation [530,531]. In an RCT of overweight hyperuricemic adults, neither allopurinol nor probenecid could lower blood pressure [532]. A decrease of uric acid by allopurinol was linked to a rise in serum calcitriol [533]. A recent Cochrane meta-analysis pointed out that there might be few data suggesting a positive impact of uric acid lowering on the progression of kidney dysfunction in the short term (1 year) but not in the long term, whereas not enough data was present in favor of an effect on other endpoints (blood pressure, death, proteinuria, cardio-vascular markers) [534]. Overall, quality of studies was low. Finally, an umbrella review including more than 100 articles reporting on meta-analyses of randomized and observational trials and Mendelian randomization studies, considering 136 unique health outcome parameters, including CKD incidence or progression and mortality in CKD, showed no convincing evidence for a clear association between uric acid levels and outcomes, apart from the occurrence of gout and uric acid kidney stones [535]. 

Thus, in spite of some arguments in favor of the toxicity of uric acid, not all data on its clinical impact and the effect of pharmaceutical reduction of its concentration are consistent, especially in RCTs applying uric acid decreasing drugs. Most, if not all, studies were of limited extent and essentially based on surrogate endpoints. All meta-analyses were fairly inconclusive for progression of CKD or hard outcomes in CKD. A phase 3 placebo-controlled RCT with renal function as the primary endpoint is ongoing [536]. In addition, the main present interventions to decrease uric acid pharmaceutically, allopurinol or febuxostat, have by themselves, a sizeable complication profile (bone marrow suppression, severe cutaneous adverse reactions, hepatotoxicity) so that studies suggesting an effect should rather be considered as a proof of concept than as an incentive for treatment, especially in asymptomatic hyperuricemia. A further note of caution derives from a recent RCT comparing febuxostat to allopurinol, in which febuxostat showed a higher risk for all cause and cardio-vascular death for a similar effect on gout flares, in spite of lower serum uric acid levels [537]. More than half of the patients had CKD stage G 3 [537]. Another urate lowering drug, the uricosuric agent lesinurad, was associated with a dose dependent decrease in serum urate but an increase in nephrotoxicity and mortality with the highest dose [538] which was subsequently not marketed. 

#### 4.3.6. Conclusion

Use of specific drugs to decrease the activity of uremic toxins in CKD has been deceiving in general (Table 6). Further and hard outcome studies are needed to sort out whether some suggestive data of a benefit (homocysteine, possibly uric acid) are corroborated in larger studies. Possibly patho-physiology and retention pattern of CKD are too complex and multifactorial to allow therapies affecting one single retention solute to revert the deleterious outcomes of CKD. 

## 5. General Conclusions

Patients with CKD suffer from a disorder with complex patho-physiology with intertwined mechanisms, whereby, especially inflammation, hypertension, hyperglycemia, metabolic changes and disturbed bone homeostasis lead to complications and high mortality rate. Pharmaceutical management cannot always be based on high-level evidence, due to the difficulties in recruiting patients with sufficiently homogeneous background regarding the primary disease, metabolic features and response to the uremic syndrome. As a result, specific therapeutic recommendations are often based on an amalgam of high and low levels of evidence and uremia-related patho-physiologic reasoning. Therapeutic approaches cannot always be extrapolated from the general population, since in a number of studies, beneficial interventions in the general population had a different effect in CKD as with intensive glycemic control or anticoagulation. Treatment of one aspect of the uremic syndrome might also exacerbate other damaging pathways as experienced, e.g., with bardoxolone. Possibly, in future studies, to cope with the high mortality of CKD, considering a more holistic therapeutic approach may turn out to be more useful rather than pursuing one single factor. Therapeutic approaches should also focus on additional outcomes beyond mortality, especially standardized quality of life. Consultation with patients to understand what is important to them might be useful for defining patient-related outcomes. 

**Figure 2:**
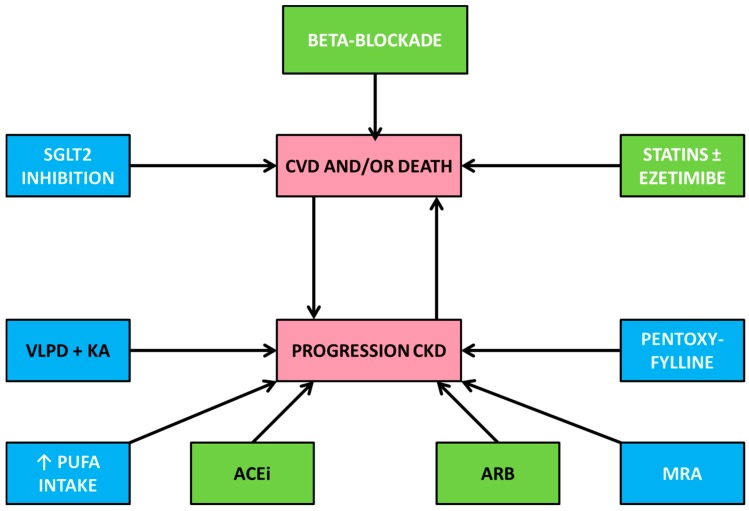
Main interventions with the potential to reduce cardio-vascular risk. Pink background: outcomes to be modified; green background: strong arguments in favor of a benefit (corresponding to +++ in the tables); blue background: suggestive arguments in favor of benefit (corresponding to ++ in the tables). White characters: point to a drawback explained below. SGLT2: sodium glucose transporter 2; CVD: cardio-vascular disease; VLPD: very low protein diet; KA: ketoanalogues; ACEi: angiotensin converting enzyme inhibitors; ARB: angiotensin receptor blockers; MRA: mineralocorticoid receptor antagonism. Drawbacks—beta-blockade: evidence restricted to CKD populations with heart failure; SGLT2 antagonism: activity proven only for diabetic nephropathy and not proven in studies specifically designed for chronic kidney disease; statins ± ezetimibe: not to be started in patients on dialysis; pentoxyfilline: only proven for add-on therapy to RAS inhibition; ↑ PUFA intake: based on a mix of data considering either fish intake or real PUFA administration; mineralocorticoid antagonism: add-on therapy to ACEi or ARB, risk of hyperkalemia, and renal benefit only proven for albuminuria/proteinuria. Several of these interventions may have an impact not only on the outcome indicated by an arrow, but also on the other outcome, be it with less strong supportive data—this is the case for SGLT2 inhibition, statins ± ezetimibe, very low protein diet + ketoanalogues, ↑ PUFA intake, ACEi, ARB, and mineralocorticoid receptor antagonism. For details and references, see Table 2 and Table 4, Table 5, Table 6. The figure does not take into account health economic aspects.

Although several of the interventions discussed in this publication are not well-supported by evidence-based studies to accept them as standard in all CKD patients, other approaches are supported by more convincing results (Figure 2). The comprehensive tables summarizing available data may be of help in guiding the pharmacological treatment of CKD and in preventing cardio-vascular disease, either directly or indirectly via preserving kidney function. Although some of the evidence is fairly straightforward, it is conceivable that most of the measures proposed in this article are still insufficiently implemented [539], such as for anti-hypertensive treatment. Our summary also may help to define which aspects might be promising or where additional studies might be helpful. Only a few therapies, such as statins alone or combined with ezetemibe, ACE inhibition, angiotensin receptor blockade, beta blockade and maybe add-on aldosterone antagonism and pentoxifylline (Table 4, part B and Table 5, part C) can now be considered as having established benefit either on cardio-vascular outcomes, the progression of CKD or both. Other therapies such as SGLT-2 inhibition are now also supported by fairly solid evidence (Table 5, part B) but would benefit from studies with primary renal outcomes specifically developed to answer CKD-related questions. Some novel originally promising therapies such as NFκB inhibition, endothelin receptor A blockade and AST-120 (Table 5, parts D and I and Table 6) subsequently gave disappointing results and may need the definition of the populations with real benefit and reanalysis in those populations. A number of established drugs used for other indications, e.g., vitamin D analogues and metformin (Table 5, parts A and B) may find a new destination in the treatment for CKD patients if their supposed benefit is confirmed in hard outcome studies. More reserve is needed for several drug groups or therapies that have been tested with often debatable success or not been tested at all in RCTs in CKD, such as intensive hypertension control, phosphate binding, calcimimetics, and homocysteine and uric acid lowering (Table 4, Table 5, parts B, D and E, and Table 6), unless novel evidence is convincing enough to generate a mind shift. Available data suggest that correction of hypomagnesemia may offer some interesting features, but this has never been tested thoroughly with sufficiently powered RCTs. Finally, this article also mentions promising novelties such as aryl hydrocarbon receptor blockade, but these would need extensive clinical exploration before they come to pass. 

A major problem with the appearance on the horizon of novel drugs is their substantial cost either for the patient, society, or both. In the area of CKD, there is a striking lack of health economic analyses, and effort should be enhanced to broaden this knowledge. Such analyses should be undertaken by independent institutions, not by the interested pharmaceutical industry itself. Regulatory decision making for drug reimbursement should be based on robust evidence and reliable socio-economic benefit analysis. Pharmaceutical companies should somehow be convinced by the medical communities and the regulatory bodies to follow an ethical code considering their role in society and not base their activities on financial interest only. For the time being, a health economic benefit vs. no treatment was demonstrated to a certain extent only for ACE inhibition, angiotensin receptor blockade, and cholesterol lowering therapies (Table 4, part B and Table 5, part C).

Finally, the areas of lifestyle measures and diet are underexplored and underexploited. In spite of suggestive data of small or short-term studies, there is an urgent need for assessments over longer observation periods, in larger numbers of patients and on hard end points because these approaches, in general, are not expensive for society. However, their implementation may necessitate a shift of paradigm, with more focus on education and interaction with the patient and the general population, and less on drug prescription. Awaiting more solid evidence, such measures could already be implemented now, whereby demographic evolution might already answer a number of pending questions in this respect. According to our analysis, two interventions, very low protein diet plus ketoanalogues and increasing intake of polyunsaturated fatty acids, received somewhat more robust support, but extended analysis is still needed.

Given the persisting high cardio-vascular mortality in CKD, we hope that the kaleidoscopic overview offered in this publication may be helpful in choosing the interventions that are likely to counter this problem and which ones are worth further investment in future analysis.

## Figures and Tables

**Table 1:** Chronic kidney disease (CKD) and its different stages.

Stage	GFR ^a^	Definition
G1	>90 ^b^	Normal or increased kidney function and urine abnormalities (e.g., hematuria from presumed or proven renal origin or albuminuria ^c^), proven structural kidney abnormalities detected by imaging, genetic trait for kidney disease, electrolyte abnormalities from renal tubular dysfunction, renal histological abnormalities or history of kidney transplantation
G2	60–89	Mild reduction in kidney function and urine abnormalities (e.g., hematuria from presumed or proven renal origin or albuminuria ^c^), proven structural kidney abnormalities detected by imaging, genetic trait for kidney disease, electrolyte abnormalities from renal tubular dysfunction, renal histological abnormalities or history of kidney transplantation
G3a G3b	45–59 30–44	Moderately reduced kidney function
G4	15–29	Severely reduced kidney function
G5	<15	Very severe kidney failure or ESKD, on dialysis or not

GFR: glomerular filtration rate (mL/min/1.73 m^2^); ESKD: end stage kidney disease. ^a^ GFR can be directly measured [clearance of e.g., iohexol, inulin, iothalamate, ethylene diamino acetic acid (EDTA)], or calculated based on serum markers such as creatinine and/or cystatin C, gender and ethnicity (estimated GFR—eGFR); ^b^ as GFR tends to decline with age (approximately 1 mL/min/1.73 m^2^ per year from the age of 40 on), the average reference value is below 90 mL/min/1.73 m^2^ from the age of 60 on; ^c^ albuminuria is classified as moderately increased (category A2, 30–300 mg/g creatinine or 3–30 mg/mmol) and severely increased (category A3, >300 mg/g or >30 mg/mmol) and each of these albuminuria categories increases the risk of progressive CKD and all cause and cardiovascular mortality.

**Table 2:** Summary of available data on lifestyle measures and diet.

	Cardiovascular Events or Mortality	References	Development of Progression of CKD ^a^	References	Health Economic Impact ^b^	References
**A.** **Lifestyle modifications**						
Smoking cessation	+	**[28]**,**[29]**	+	**[21]**,**[22]**,**[23]**,**[24]**,**[25]**,*[29]*	+	**[30]**,**[31]**,*[32]*,*[33]*,**[34]**
Exercise	+	**[46]**,**[47]**	+	**[46]**,**[47]**	+	**[40]**,**[41]**,**[42]**
Reduction of obesity			+	*[63]*,**[64]**,**[65]**,**[66]**,**[67]**	+	**[53]**,**[54]**,**[55]**,*[33]*
**B.** **Dietary measures**						
Low protein diet			+	*[76]*,*[77]*,*[78]*,*[80]*,**[84]**,**[85]**,*[86]*,*[87]*		
Very low protein diet + KA			++	**[81]**,**[84]**,**[86]**		
Low Na^+^ diet			+	**[90]**,*[112]*,*[113]*, **[115]** ^c^, **[116]** ^c^,**[117]** ^c^		
K^+^-rich diet ^d^			+	**[113]**,**[47]**		
P-restriction	+	*[133]*,**[134]**	+	**[129]** ^e^,**[130]** ^e^,**[131]**,**[132]** ^e^		
Enhanced PUFA-intake	+	**[138]**,**[139]**,**[140]** ^f^,**[141]**,**[142]**,*[143]*	++	**[144]**,**[145]**		
Reduced glucose intake			+	**[146]**,**[148]**	+	**[150]**
Reduced AGE intake	+	**[154]**,*[155]*	+	**[158]**		
Correction intestinal dysbiosis			+	**[164]**,**[166]**		

++: 2–4 randomized controlled trials and/or at least 1 meta-analysis; +: indirect, observational or contradictory data or 1 RCT. Blank space means that insufficient data were retrieved to allow a judgment; factors discussed in specific sections of the text about which overall insufficient evidence could be collected were omitted from the table (improving environmental factors; reducing fructose intake). ^a^ decrease of GFR or eGFR, or rise in serum creatinine or albuminuria; ^b^ data may be based on studies in the general population; ^c^ if combined to inhibition of the renin angiotensin system (RASi); ^d^ only up to CKD stage G2 and in patients who are not prone to hyperkalemia; ^e^ studies deal with serum phosphate rather than with phosphate intake; ^f^ studies deal with fish intake rather than with polyunsaturated free fatty acid (PUFA)-intake per se. CKD: chronic kidney disease; KA: ketoanalogues; Na^+^: sodium; K^+^: potassium; P: phosphorus; AGE: advanced glycation end products. References—bold: positive effect; italics: negative or no effect; underscore: randomized controlled trial or meta-analysi.

**Table 3:** Integrated optimal nutritional track of CKD patients.

	CKD 3–5 ND	Hemodialysis	Peritoneal Dialysis	Transplantation First 3 Months	Transplantation > 3 Months
Target protein intake (g/kg/d)	0.6–0.8 (or less + KA)	1.0–1.2	1.0–1.2	1.4	0.6–0.8
Salt intake (mg/d) *	<6000	<5000	<5000	<6000	<6000
Potassium intake (mg/d)	2500	2500	2500	Free **	Free **
Phosphorus intake (mg/d)	<800	<1000	<1000	Free	<800

CKD: chronic kidney disease; ND: not on dialysis; KA: keto-analogues *: except in case of salt loosing nephropathy; **: except in case of hyperkalemia (>5.5 mmol/L).

**Table 4:** Summary of available data on antihypertensive treatment.

	Cardiovascular Events or Mortality	References	Development of Progression of CKD ^a^	References	Health Economic Impact	References
**A.** **Intensity antihypertensive therapy**						
Intensive treatment non-diabetic CKD ^b^	+	*[172]*,*[173]*,**[175]**,**[177]**,[183],*[184]*,**[185]**	+	*[172]*,*[173]*,*[175]*,[177],**[182]** ^c^,*[183]*,*[184]*		
Standard treatment ESKD (hemodialysis) ^d^	+	**[190]**,**[191]**				
**B.** **Specific antihypertensive approaches**						
ACEi	+	*[201]*,*[202]*,**[203]**,*[211]*,**[212]**,*[213]*	+++	**[197]**,**[198]**,**[199]**,**[200]**,**[201]**,**[202]**,**[203]**,*[211]*,**[212]**,*[213]*	++	**[217]**,**[218]**,**[219]**
ARB	+	*[206]*,*[211]*,**[212]**,*[213]*	+++	**[121]**,**[198]**,**[204]**,**[205]**,**[206]**,**[207]**,**[206]**,**[211]**,**[212]**,*[213]*	+	**[220]**
ACEi + ARB ^e^	+	**[215]**	+	**[211]**,*[214]*		
Aldosterone antagonists	+	**[215]**,**[233]**	++ ^f^	**[226]**,**[227]**,**[228]**,**[229]**,**[230]**,**[231]**		
Beta blockers ^g^	+++	**[255]**,**[256]**,**[257]**,**[258]**,**[259]**				

+++: at least 4 randomized controlled trials and/or 2 meta-analyses; ++: 2–4 randomized controlled trials and/or at least 1 meta-analysis; +: indirect, observational or contradictory data or 1 RCT; blank space means that insufficient data were retrieved to allow a judgment; factors discussed in specific sections of the text about which overall insufficient evidence could be collected were omitted from the table (intensive antihypertensive treatment of diabetic CKD; the combination of inhibitors of the renin angiotensin system (RAS) and intestinal potassium binders). ^a^: decrease of GFR or eGFR, or rise in serum creatinine or albuminuria; ^b^: gradual decrease preferred, follow-up kidney function needed; ^c^: in case of proteinuria > 1g/d; ^d^: blood pressure assessment by preference not to be based on pre-dialysis or intra-dialysis blood pressure; ^e^: risk of hyperkalemia; ^f^: only decrease in albuminuria; often at the expense of hyperkalemia and sometimes of a decline in eGFR; ^g^: in heart failure. CKD: chronic kidney disease; ESKD: end stage kidney disease; ACEi: angiotensin converting enzyme inhibitor; ARB: angiotensin receptor blocker. References—bold: positive effect; italics: negative or no effect; underscore: randomized controlled trial or meta-analysis.

**Table 5:** Summary of available data on other interventions than antihypertensive treatment.

	Cardiovascular Events or Mortality	References	Development or Progression of CKD ^a^	References	Health Economic Impact	References
**A.** **Glycemia control**						
Intensive glycemia control			+ ^b^	**[260]**,**[261]**,**[262]**,**[263]**,**[264]**,**[265]**		
SGLT-2 inhibitors	++	**[274]**,**[276]**,**[281]**,**[282]**	+	**[274]**,**[275]**,**[276]**,**[277]**,**[278]**,**[279]**		
DDP-4 inhibitors			+ ^c^	**[287]**		
GLP-1RA	+	**[291]**	+ ^c^	**[288]**,**[291]**		
Metformin	+	**[294]**,**[295]**,**[297]**	+	**[294]**		
**B.** **CKD-MBD**						
Phosphate binders (aggregated) ^d^	+	**[308]**,**[309]**,*[312]*,*[313]*,*[314]*				
Non-calcium phosphate binders ^e^	+	*[323]*,**[324]**,**[325]**,**[326]**,*[328]*,**[329]**,**[314]**,**[331]**,**[332]**,*[330]*			+	*[334]*,**[335]**,**[336]**,**[337]**,**[338]**
Calcimimetics	+	**[341]**,*[343]*,**[345]**,*[346]*				
Parathyroidectomy	+	**[347]**				
Calcitriol and vitamin D analogs ^f^			+ ^c^	**[352]**,**[353]**,**[354]**,*[355]*		
**C.** **Dyslipidemia**						
Statins ± ezetemibe (non-dialysis)	+++	**[360]**,**[361]**,**[362]**,**[363]**,**[364]**,**[365]**	+ ^c^	**[361]**,**[362]**,*[366]*	+	**[378]**,**[379]**
**D.** **Inflammation**						
NFκB inhibition ^g^			+	**[389]**,*[390]*		
CCR2 inhibition ^h^			+ ^c^	**[395]**,**[397]**		
Pentoxifylline ^i^		**[398]**	++	**[398]**,**[399]**,**[400]**		
**E.** **Hypomagnesemia**						
Correction of hypomagnesemia	+	**[418]**,**[419]**,**[420]**,**[422]**	+	**[417]**		
**F.** **Metabolic acidosis**						
Acidosis correction (mainly bicarbonate)			+	**[427]**,**[428]**		
**G.** **Thrombophilia**						
Antiplatelet therapy	+ ^k^	*[451]*,*[452]*,**[454]**				
Vitamin K antagonists ^j^	+ ^k^	*[455]*,*[456]*,**[457]**				
**H.** **Kidney fibrosis**						
Pirfenidone			+	**[476]**,*[477]*		
**I.** **Protein-bound toxin retention**						
AST-120 sorbent	+	**[494]**	+	**[485]**,**[486]**,**[487]**,**[488]**,[*490]*,**[491]**,**[492]**,*[493]*,**[494]**,**[495]**	+	**[489]**

+++: at least 4 randomized controlled trials and/or 2 meta-analyses; ++: 2–4 randomized controlled trials and/or at least 1 meta-analysis; +: indirect, observational or contradictory data; blank space means that insufficient data were retrieved to allow a judgment; factors discussed in specific sections of the text about which overall insufficient evidence could be collected were omitted from the table (calcium-containing phosphate binders vs. no treatment; neutralization of fibroblast growth factor-23 (FGF-23); statins ± ezetimibe in dialysis patients; blockade of PCSK9 and ANGPTL3; periodontal care; aryl hydrocarbon receptor (AhR) blockade). ^a^: decrease of GFR or eGFR, or rise in serum creatinine or albuminuria; ^b^: high risk for hypoglycemia and negative hard outcomes, especially in CKD—apply in function of the clinical condition of the patient; ^c^: only albuminuria/proteinuria—score downgraded because no evidence for effect on progression of CKD; ^d^: calcium and non-calcium phosphate binders together vs. placebo; ^e^: mainly sevelamer, but also lanthanum and iron-containing phosphate binders vs. calcium phosphate binders; ^f^: only paricalcitol; ^g^: phase 3 study prematurely discontinued because of higher cardiovascular event rate with study drug (bardoxolone); ^h^: add-on to RAS-inhibition in diabetic nephropathy; ^i^: add-on to RAS inhibition; ^j^: often at the expense of more bleeding. CKD: chronic kidney disease; SGLT-2: sodium-glucose cotransporter-2; DDP-4: dipeptidyl peptidase-4; GLP-1 RA: glucagon-like peptide-1 receptor agonist; NFκB: nuclear transcription factor kappa B; CCR2: CC chemokine receptor 2; RAS: renin angiotensin system. References—bold: positive effect; italics: negative or no effect; underscore: randomized controlled trial or meta-analysis.

**Table 6:** Summary of available data on drugs affecting the concentration of specific uremic toxins.

	Cardiovascular Events or Mortality		Development of Progression of CKD ^a^		Health Economic Impact ^b^
Advanced glycation end product reduction			+	**[497]**	
Cytokine antagonism			+ ^b^	**[503]**	
Endothelin A receptor blockade			+ ^c^	**[505]**,**[506]**,**[507]**,**[509]**	
Homocysteine lowering	+	*[515]*,*[516]*,*[517]*,*[518]*,**[521]**,**[522]**	+	**[520]**	
Uric acid lowering	+	**[524]**,**[530]**,**[531]**,*[532]*,*[534]*,*[535]*	+	**[530]**,**[531]**,*[534]*,*[535]*	

+: indirect, observational or contradictory data or 1 RCT. ^a^: decrease of GFR or eGFR or rise in serum creatinine or albuminuria; ^b^: in rheumatoid arthritis; ^c^: decrease in proteinuria but at the expense of more fluid retention inducing higher mortality.
